# Airport emission particles: exposure characterization and toxicity following intratracheal instillation in mice

**DOI:** 10.1186/s12989-019-0305-5

**Published:** 2019-06-11

**Authors:** Katja Maria Bendtsen, Anders Brostrøm, Antti Joonas Koivisto, Ismo Koponen, Trine Berthing, Nicolas Bertram, Kirsten Inga Kling, Miikka Dal Maso, Oskari Kangasniemi, Mikko Poikkimäki, Katrin Loeschner, Per Axel Clausen, Henrik Wolff, Keld Alstrup Jensen, Anne Thoustrup Saber, Ulla Vogel

**Affiliations:** 1 National Research Centre for the Working Environment, Lersø Parkallé 105, DK-2100 Copenhagen, Denmark; 2 National Centre for Nano Fabrication and Characterization, Technical University of Denmark, Fysikvej, Building 307, DK-2800 Kgs Lyngby, Denmark; 3 FORCE Technology, Park Allé 345, 2605 Brøndby, Denmark; 4 Aerosol Physics, Laboratory of Physics, Faculty of Natural Sciences, Tampere University of Technology, PO Box 527, FI-33101 Tampere, Finland; 5 National Food Institute, Research Group for Nano-Bio Science, Technical University of Denmark, Kemitorvet 201, DK-2800 Kgs Lyngby, Denmark; 6 Finnish Institute of Occupational Health, P.O. Box 40, FI-00032, Työterveyslaitos, Helsinki, Finland; 7 Department of Health Technology, Technical University of Denmark, DK-2800 Kgs Lyngby, Denmark

**Keywords:** Airport, Exposure risk, Jet engine emission, Jet engine particle, Occupational exposure

## Abstract

**Background:**

Little is known about the exposure levels and adverse health effects of occupational exposure to airplane emissions. Diesel exhaust particles are classified as carcinogenic to humans and jet engines produce potentially similar soot particles. Here, we evaluated the potential occupational exposure risk by analyzing particles from a non-commercial airfield and from the apron of a commercial airport. Toxicity of the collected particles was evaluated alongside NIST standard reference diesel exhaust particles (NIST2975) in terms of acute phase response, pulmonary inflammation, and genotoxicity after single intratracheal instillation in mice.

**Results:**

Particle exposure levels were up to 1 mg/m^3^ at the non-commercial airfield. Particulate matter from the non-commercial airfield air consisted of primary and aggregated soot particles, whereas commercial airport sampling resulted in a more heterogeneous mixture of organic compounds including salt, pollen and soot, reflecting the complex occupational exposure at an apron. The particle contents of polycyclic aromatic hydrocarbons and metals were similar to the content in NIST2975. Mice were exposed to doses 6, 18 and 54 μg alongside carbon black (Printex 90) and NIST2975 and euthanized after 1, 28 or 90 days. Dose-dependent increases in total number of cells, neutrophils, and eosinophils in bronchoalveolar lavage fluid were observed on day 1 post-exposure for all particles. Lymphocytes were increased for all four particle types on 28 days post-exposure as well as for neutrophil influx for jet engine particles and carbon black nanoparticles. Increased *Saa3* mRNA levels in lung tissue and increased SAA3 protein levels in plasma were observed on day 1 post-exposure. Increased levels of DNA strand breaks in bronchoalveolar lavage cells and liver tissue were observed for both particles, at single dose levels across doses and time points.

**Conclusions:**

Pulmonary exposure of mice to particles collected at two airports induced acute phase response, inflammation, and genotoxicity similar to standard diesel exhaust particles and carbon black nanoparticles, suggesting similar physicochemical properties and toxicity of jet engine particles and diesel exhaust particles. Given this resemblance as well as the dose-response relationship between diesel exhaust exposure and lung cancer, occupational exposure to jet engine emissions at the two airports should be minimized.

**Electronic supplementary material:**

The online version of this article (10.1186/s12989-019-0305-5) contains supplementary material, which is available to authorized users.

## Background

Airport personnel are at risk of complex occupational exposures originating from many sources, including combustion particles from jet engines and diesel-fueled handling vehicles. Exposure to ultrafine particles (UFP, diameter ≤ 100 nm) from combustion exhaust has consistently been associated with a wide range of health risks [1, 2]. Diesel engine exhaust and diesel exhaust particles, which are a major component of ultrafine particles (UFP) in urban aerosols, have been classified as carcinogenic to humans (group 1) by the International Agency for Research on Cancer (IARC) [3] and cause lung cancer, systemic inflammation and inflammatory responses in the airways [4].

There is increasing awareness of the potential health risk due to occupational fuel combustion exposures at airports and studies of airport personnel health and exposure are accumulating. A large cohort study following 69,175 workers at Copenhagen Airport from 1990 to 2012 included data such as lifestyle characteristics, work tasks, and air pollution. By linkage to health registers this cohort will be monitored for incidence of cardiovascular diseases, cancer, and pulmonary diseases [5]. An Italian study reported DNA aberrations in airport staff (sister chromatid exchange and total structural chromosomal changes in lymphocytes and exfoliated buccal cells) with increased tail moment in the comet assay compared to unexposed controls [6]. Evaluation of airport workers in Turkey [7] and at an American aircraft equipment military station [8] also showed a significant increase in the frequency of sister chromatid exchange in the exposed workers. Recently, it was shown that 2 hours of normal breathing in a high-concentration airport-particle zone downwind of Los Angeles airport increased the acute systemic inflammatory cytokine IL-6 of non-smoking adults with asthma [9]. However, studies assessing the potential health hazards of jet engine particles without confounding life style factors are limited. A study of the jet fuel JP-8, where mice were exposed to vapor and aerosol exposure, reported potential effects on lung surfactant [10].

Studies of the hazard potential of environmental exposures benefit from inclusion of well-characterized control particles or standard reference materials (SRM) because this allows comparison of the studied exposures with exposures to particles of well-known toxicity. Diesel exhaust particles have been extensively evaluated in animal studies and in humans [11–14] and are therefore suitable as benchmark particles. The standard reference material SRM 2975 (forwardly referred to as NIST2975) from the National Institute of Standards and Technology (NIST, Gaithersburg, MD, USA) is a sample of diesel exhaust particles collected from an industrial fork lift [15] which contains low levels of polycyclic aromatic hydrocarbons (PAH). The NIST SRM 1650b (forwardly referred to as NIST1650) is diesel particles collected from a heavy duty diesel truck engine and contain more PAH compared to NIST2975. The pigment carbon black has been classified as possibly carcinogenic to humans [3]. Carbon black Printex 90 (CB) is black pigment used in printing ink consisting of carbon nanoparticles with very low levels of contaminants. We previously showed that intratracheal instillation with NIST1650 and CB induce pulmonary acute phase response, neutrophil influx, and genotoxicity [16–22]. Genotoxicity was observed even at very low doses of CB [23]. The potential similarity of jet engine exhaust particles with diesel exhaust particles and carbon nanoparticles, such as CB, warrants a hazard risk assessment of jet engine exhaust particles.

The purpose of the current study was to assess the pulmonary toxicity of airplane emissions in mice and to compare this with reference particles of known toxicity. We characterized the exposure at a commercial airport and at a non-commercial airfield and characterized the physical/chemical properties of collected particles from both locations. Finally, we assessed the acute phase response, inflammation, and genotoxicity following pulmonary exposure to these two different samples of airplane emissions at three different dose levels and three different time points in mice (Table 1 gives an overview of the data and relevant figures). Standard reference materials with known toxicity, namely diesel particle NIST2975 and carbon black Printex90 (CB) nanoparticles as well as available published data on NIST1650 [23] were included in the study for comparison.

**Table 1:** Overview of samples

*Particle type*	*Measurement*	*Instruments/Method*	*Relevant figures*	
Non-commercial airfield particles (JEP)	*Exposure characterization*	1 ELPI	Figure 1:	Exposure characterization
		4 DISCminis	Table 2:	Exposures and doses
		1 NanoScan	Additional File 1: Figure S1 A:	Position of instruments Jet engine test facility
		1 OPC	Additional File 1: Figure S1 B:	
	*Background characterization*	Micro INertial Impactor (MINI)	Results not shown	
	*Emission characterization*	Micro INertial Impactor (MINI)	Additional File 1: Figure S1 C:	Description of impacted aerosols and TEM images
	*Particle collection for physical and chemical characterization and mouse instillations*	Electrostatic precipitator	Table 3:	PAH contents Metal contents
			Table 4:	
	*JEP particles suspended in instillation vehicle*	TEM (dropcast)	Table 5:	Size distribution
			Additional File 1: Figure S1 D:	DLS figures
			Fig. 2:	SEM images
			Additional File 1: Figure S1E:	Elemental composition by EDS analysis
Commercial airport particles (CAP)	*Exposure characterization*	4 DISCminis	Figure 1:	Exposure characterization
		1 NanoScan	Additional File 1: Figure S1 A:	Position of instruments
		1 OPC		
	*Emission characterization*	Micro INertial Impactor (MINI)	Additional File 1: Figure S1 C:	Description of impacted aerosols and TEM images
	*Particle collection for physical and chemical characterization and mouse instillations*	Electrostatic precipitator	Table 3:	PAH contents
			Table 4:	Metal contents
	*CAP particles suspended in instillation vehicle*	TEM, dropcast	Table 5:	Size distribution
			Additional File 1: Figure S1 D:	DLS figures
			Figure 2:	SEM images
			Additional File 1: Figure S1 E:	Elemental composition by EDS analysis
Mouse instillations of JEP and CAP	*Lung pathology*	Histology	Figure 3:	Histopathology of lung sections
	*Cellular composition in the lungs*	Broncho-alveolar lavage (BAL)	Table 6:	BAL fluid cell composition
			Figure 4:	Dose-response relationship of instilled particles
			Figure 5:	Neutrophil influx
			Additional File 2: Figure S2 A:	Scatter plots of cellular influx Eosinophil influx
	*Serum Amyloid A levels in tissues*	mRNA expression	Figure 6:	SAA day 1
			Additional File 2: Figure S2 B:	SAA day 28 and 90
	*DNA damage*	DNA strand breaks (Comet Assay)	Figure 7:	Tail Length
			Additional File 2: S2C:	% DNA in Tail and data table

## Results

### Aerosols

#### Particle exposure characterization at a non-commercial airfield

**Fig. 1:**
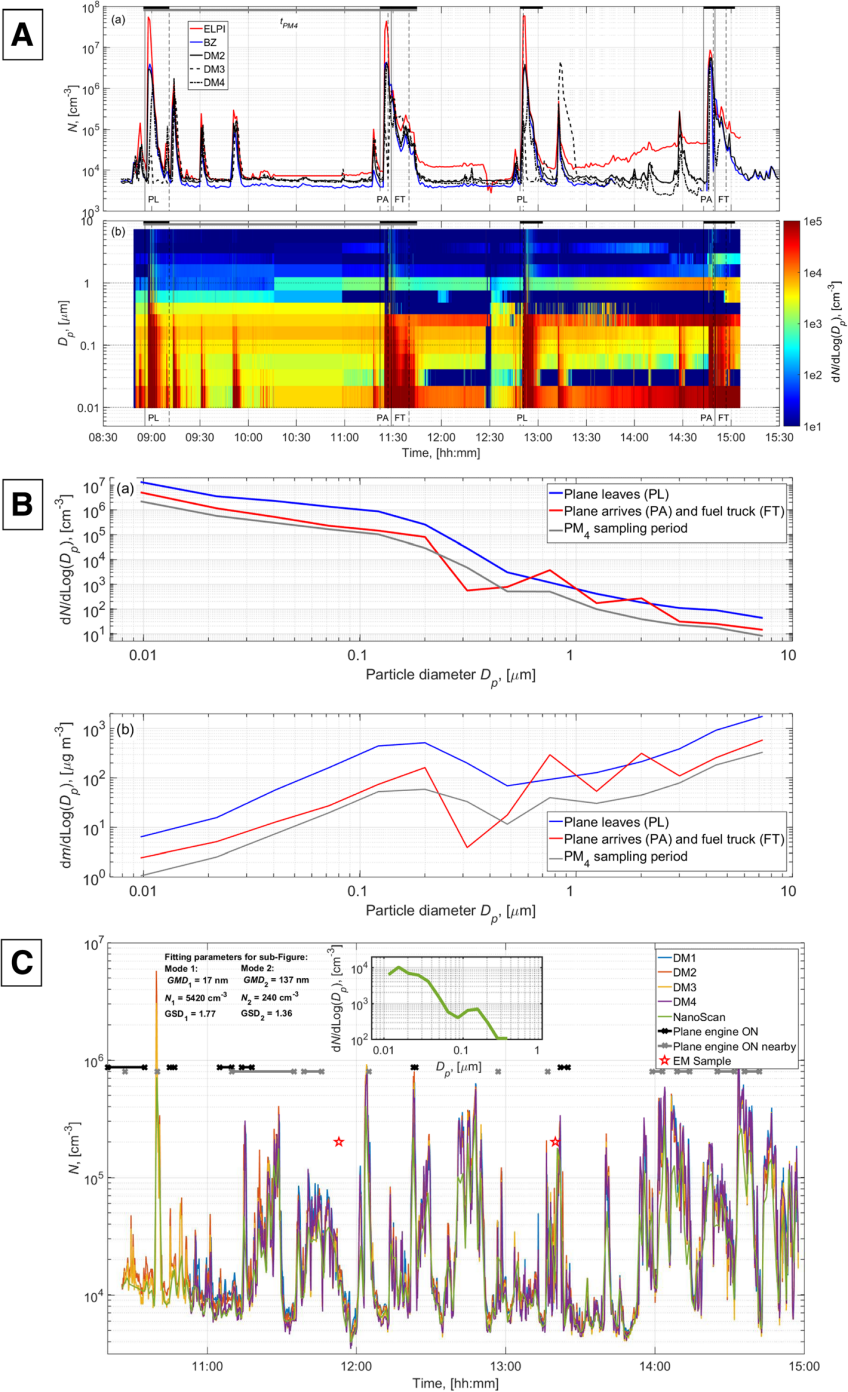
Particle concentrations measured inside a jetfighter shelter at a non-commercial airfield (**a** and **b**) and at a non-commercial airport (**c**) (see also Additional file S1 A). **a**: Total particle number concentrations (a) and particle number size distribution time series (b) inside the shelter measured during jetfighter leaving the shelter (PL), arriving at the shelter (PA), and fuel truck (FT) fueling the plane. The vertical solid and dashed black lines show when the jet engine is started or fuel truck arrives to the shelter and when the engine is switched off or fuel truck leaves the shelter. Horizontal thick black line shows the averaging period to calculate exposure and dose levels presented in Table 2. Particle sampling time for one flight cycle (tPM4) for mass fraction smaller than 4 μm (mPM4) gravimetric analysis is shown with gray vertical bar. **b**: Average particle number (a) and mass (b) size distributions. **c**: Total particle number concentrations measured at a commercial airport (CAP). The inserted sub-figure shows the average particle size distribution measured by the NanoScan during the measurement period

Two full cycles representative of a normal workflow of Plane Leaving (PL), Plane Arriving (PA) and refueling by a Fuel Truck (FT) were recorded in a jet shelter using both stationary and portable devices (see Additional file 1: Figure S1 A for outline). During the main combustion events of PL and PA, the instruments reached their upper detection limits of 10^6^ (DiSCmini) and 10^8^ (ELPI) particles/cm^3^. Importantly, this included the breathing zone monitor of the airfield personnel. Overall, but especially in main peaks, the ELPI detected mainly particles under 500 nm (Fig. 1a). The number size distributions during PL, PA, and FT suggested that the prevalent particle sizes were probably below the detection limit of the ELPI, suggesting that the jet engine combustion particles are below 10 nm in aerodynamic diameter (Fig. 1b). This was similar to the particle number and size distributions in measurements of jet engine exhaust conducted in a jet engine test facility (see Additional file 1: Figure S1 B). In the size-resolved mass distributions for PL, PA, and FT, there was a mode around 150–200 nm and the remaining mass was allocated with larger particle sizes up to the detection limit of 10 μm (Fig. 1b). The particle concentrations measured by the four DiSCmini devices followed the same event-specific trends with a slightly lower background signal for the personal monitor. The two events of PA (large peak) and FT arrival (subsequent shoulder) were not fully discernable and have therefore been combined into a single event in the analysis. The event-related air concentrations and the corresponding predicted lung deposition are shown in Table 2.

**Table 2:** Average exposures and doses of jetfighter personnel at a non-commercial airfield

Event	*t*, [min]	*n*, ×10^6^ [cm^−3^]	*m*, [μg m^− 3^]	*m*_*PM4*_, [μg m^− 3^]	*DR*_*N*_, × 10^10^ [min^− 1^]	HA, n[%]	TB, n[%]	AL, n[%]	*DR*_*m*_, [μg min^− 1^]	HA, m[%]	TB, m[%]	AL, m[%]	Particles [× 10^12^]/ Event	Mass [μg]/ Event
PL	15.1	7.7	1086	537	15	21.2	27.2	51.6	18.7	84.6	4.7	10.7	2.26	280
PA + FT	21.3	2.67	410	228	5.4	21.7	27.7	50.7	7	83.6	4.9	11.5	1.15	150
t_PM4_	170	1.22	194	89	2.4	21.4	27.4	51.3	3.5	85.8	4.6	9.6	4.12	600

Average exposures and doses during Plane Leaving (PL), Plane Arrival and fueling the plane (PA + FT combined), and over one flight cycle (t_PM4_). From left to right: average event time (*t*) in minutes, average particle number concentration (*n*), mass concentration (*m*) and mass fraction smaller than 4 μm (*m*_*PM4*_), inhaled number dose per minute (*DR*_*N*_), predicted fraction of particles deposited in extra-thoracic (*HA*), tracheo-bronchial (*TB*) and alveolar (*AL*) lung regions, inhaled mass dose per minute (*DRm*), predicted fraction of mass deposited in extra-thoracic (*HA*), tracheo-bronchial (*TB*) and alveolar (*AL*) lung regions, total particles per event and total mass per event

#### Collected samples of jet engine particles (JEP) from a non-commercial airfield

One JEP impactor sample was acquired when no jetfighters were running and another sample was collected near a running jet fighter in taxi, each with an electron microscopy (EM) grid installed on all three stages. The low number density observed on the grids from the background sample even after 60 s of sampling suggested that the background aerosol contained very few particles (results not shown), and therefore could be ignored when analyzing the take-off sample, which was collected for 5 s. The EM grids from the first and second stage of the take-off sample were densely populated with highly agglomerated soot particles ranging from approximately 500 nm to tens of micrometers in equivalent circular diameter (ECD). The primary soot particles were in the order of 10 to 30 nm and displayed a typical soot structure with fringes of graphene like flakes (see Additional file 1: Figure S1 C for detailed description and EM images). Due to the high particle loadings on the grids, it was not possible to determine whether the large soot agglomerates were a result of co-deposition during sampling, or whether they were airborne as agglomerates.

#### Particle exposure characterization at a commercial airport

The average DiSCmini geometric mean particle concentration and lung deposited surface area (LDSA) were 2.2 × 10^4^ cm^− 3^ (Geometric Standard Deviation (GSD) 3.6) and 24.1 cm^2^ m^− 3^ (GSD 2.6) over the measurement period, respectively. High GSD was caused by high variation in concentration levels (Fig. 1c). According to the NanoScan, the particles were mainly below 300 nm in diameter and distributed in two modes with geometric mean diameters of < 20 nm and approximately 140 nm. The measured respirable mass concentrations were all below detection limits, which corresponded to concentration levels of < 66 μg/m^3^ when an aircraft engine was running close by, < 18.6 μg/m^3^ when there was no engines running in close vicinity, and < 14 μg/m^3^ when sampled over the measurement day from 10:27 am to 3:00 pm.

#### Collected samples of commercial airport particles (CAP)

A single CAP impactor sample was collected for 30 s at the apron of the commercial airport (see Additional file 1: Figure S1 A for placement). The first stage contained many micrometer-sized particles ranging between 1 and 50 μm. The particles were mainly dominated by rectangular or square salt crystals and a few micrometer-sized particles, which appeared to be pollen. The second stage contained only very few particles, which were in the size range between 500 nm and 1 μm in ECD. The last stage of the impactor displayed an area covering approximately 12 grid squares, which was densely populated with particles. Particle sizes varied from approximately 1 μm to a few nm in ECD. Soot particles were found in three different states: as free, individual agglomerates, as well as agglomerated to other particles (e.g. larger particles, salts, and others) and associated with or captured in droplets (see Additional file 1: Figure S1 C for detailed description and EM images).

Consequently, the aerosol at the non-commercial airfield appeared to be mainly aggregates of nano-sized carbon particles (soot), whereas the aerosol at the apron of the commercial airport appeared much more complex dominated by agglomerated soot particles, salt crystals, and low volatile compounds.

### Physicochemical characterization of particles for mouse instillation

From electrostatic precipitator (ESP) sampling [24–29] at the jet shelter during a time span of approximately 15 h, 11.7 mg of JEP were collected and at the commercial airport during 4 h and 40 min, 12.3 mg particles of CAP were collected.

#### Contents of polycyclic aromatic hydrocarbons (PAH)

Analysis of the content of polycyclic aromatic hydrocarbons (PAH), showed ∑PAH concentrations (sum of 16 PAH (Table 2), ND = 0) of 0.081 mg/g in CAP and 0.05 mg/g in JEP, respectively, including contents of benzo(a)pyrene (Table 3). The PAH profiles of JEP and CAP were roughly similar. For comparison, NIST1650 and NIST2975 contained 0.22 and 0.086 mg/g, respectively, of the same PAHs.

**Table 3:** Content of 16 PAH in airport-collected particles

PAH	CAP mg/g particles	JEP mg/g particles	NIST1650B^a^ (mg/g)	NIST2975^a^ (mg/g)
Naphthalene	ND	ND	0.007(0.0004)	0.004(0.0001)
Acenaphthylene	0.009(0.0009)	0.01(0.002)	0.001(0.00004)	
Acenaphthene	ND	ND	0.0002(0.00002)	0.0005(0.00003)
Fluorene	0.001(0.00007)	0.001(0.0002)	0.001(0.00004)	0.003(0.0002)
Phenanthrene	0.008(0.0005)	0.001(0.00008)	0.07(0.004)	0.02(0,0003)
Anthracene	ND	0.001	0.008(0.0004)	0.00005(0.000002)
Fluoranthene	0.008(0.00007)	0.001(0.00008)	0.05(0.001)	0.03(0.0005)
Pyrene	0.04(0.0007)	0.007(0.00007)	0.04(0.001)	0.002(0.0002)
Benz(a)anthracene	ND	ND	0.006(0.0004)	0.001(0.00004)
Chrysene	ND	ND	0.01(0.0006)	0.006(0.0001)
Benzo(b)fluoranthene + Benzo(k)fluoranthene	0.01(0.0009)	0.02	0.009(0.0009)	0.01(0.003)
Benzo(a)pyrene*	0.005(0.0004)	0.009(0.0004)	0.001(0.0001)	0.0008(0.00004)
Dibenz(a.h)anthracene	ND	ND	0.0004(0.00008)	0.0005(0.00005)^£^
Ideno(1.2.3-cd)pyrene	ND	ND	0.004(0.0002)	0.002(0.0001)
Benzo(g.h.i)perylene	ND	ND	0.006(0.0003)	0.002(0.00009)
∑PAH	0.081	0.05	0.22	0.086

PAH was measured by GC-MS and listed as blank corrected mean values (*N* = 2) with standard deviation in parenthesis. The PAH were extracted with cyclohexane from the two water suspensions of each particle used for the instillation in mice. ND = Not Detected

^a^The highest concentrations given in the Certificate of Analysis measured by several different methods and the associated expanded uncertainty given in parenthesis. ^£^For NIST2975 the value is for Dibenz[a,h + a,c]anthracene

#### Metal contents

Semi-quantitative analysis of elemental contents by inductive coupled plasma mass spectrometry (ICP-MS) detected metals in both JEP and CAP, including lead, cobalt, nickel, arsenic, cadmium and mercury (Table 4). The metal content profiles for JEP, CAP, and NIST2975 were generally similar, but the CAP sample had the overall highest metal contents. Noteworthy, CAP contained more than three times higher concentrations of Mg, Al, Cu, Zn, Sr and Pb than JEP and NIST2975. NIST2975 contained more Zn than JEP. No metal content was detected in CB.

**Table 4:** Extracted elements from analysis of 4 mg of jet engine particles (JEP) and particles from a commercial airport (CAP)

	JEP	CAP	NIST 2975	CB	*Ref. NIST2975* ^*a*^	*Ref. CB* ^*b*^
Li	3	17	1/ND	3/ND	–	–
Mg	950	8655	291/281	ND /ND	–	–
Al	3057	9735	ND	203/0	–	–
V	6	11	5/1	ND	0.0 ± 0.0	< 1
Cr	17	146	90/102	ND	–	< 1
Mn	134	125	11/11	1 /ND	–	–
Fe	2788	5386	814/743	498/−	0.0 ± 13	11
Co	9	15	7/8	0/−	0.1 ± 0.1	< 1
Ni	200	249	55/65	0/−	0.5 ± 0.7	< 2
Cu	1147	14,884	24/5	13/3	0.9 ± 0.6	< 1
Zn	7433	31,897	13,926/17,003	ND	16 ± 4	< 2
Ga	1	3	ND	ND	–	–
As	4	5	1/2	−/1	–	< 2
Se	5	14	ND /2	ND	–	< 10
Rb	7	8	ND	ND	–	–
Sr	44	427	8/1	2/1	–	–
Ag	62	35	ND	ND	–	–
Cd	6	3	ND	ND	–	< 0.4
In	ND	1	ND	ND	–	–
Cs	1	1	ND	ND	–	–
Ba	83	103	4/ND	3/3	–	–
Hg	4	26	ND	ND	–	< 0.2
Tl	ND	1	ND	ND	–	–
Pb	100	658	97/105	ND	–	–
Bi	3	11	1/1	ND	–	–
U	ND	2	1/1	ND	–	–

Elemental concentrations are shown in units of μg/g particle (ND = not detectable). Blank concentrations were subtracted. NIST2975 and CB were analyzed in duplicates (separated by slash). ^a^Reference values from Ball et al. (2000) [30] (the study only analyzed Co, Cu, Fe, Ni, V, and Zn). Note that we extracted for significantly longer time (several days vs. overnight) and with 25% nitric acid instead of 0.1 M phosphate buffer. ^b^Reference values from the MAK-Collection for Occupational Health and Safety (written communication of unpublished data of Degussa) [31]

#### Particle size distribution in dispersion

All particles were dispersed in Nanopure water and sonicated to obtain stable dispersions [32]. The hydrodynamic number size distribution and intensity were measured by Dynamic Light Scattering (DLS) for particle concentrations of 3.24 mg/ml, 1.08 mg/ml, 0.36 mg/ml and 0.12 mg/ml, corresponding to 162, 54, 18 and 6 μg particulate matter in 50 μL instillation volume per mouse.

The average hydrodynamic particle zeta-size (Z_ave_) varied from 136 to 269 nm for CAP and from 143 to 196 nm for JEP, depending on concentration (Table 5). CB and NIST2975 formed uniform agglomerates of 50–60 nm, whereas JEP and CAP appeared more heterogeneous with particles in the Z_ave_ size range of 50–60 nm as well as larger aggregates resulting in poor poly dispersivity indices (Table 5 and Additional file 1: Figure S1 D).

**Table 5:** Size distribution in dispersion for collected airport particles, NIST2975 and carbon black Printex90 (CB)

Dose	*6 μg*		*18 μg*		*54 μg*		*162 μg*	
	*Z*_*ave*_ *(d.nm)*	*PdI*	*Z*_*ave*_ *(d.nm)*	*PdI*	*Z*_*ave*_ *(d.nm)*	*PdI*	*Z*_*ave*_ *(d.nm)*	*PdI*
CAP	136.04	0.57	168.67	0.54	269.00	0.57	N/A	N/A
JEP	143.50	0.42	142.68	0.35	196.03	0.45	N/A	N/A
NIST2975	N/A	N/A	126.40	0.15	138.52	0.23	136.62	0.22
CB	N/A	N/A	N/A	N/A	148.74	0.28	N/A	N/A

All particles were dispersed in Nanopure water. Z-Average (intensity based harmonic mean) relates to particle sizes and Polydispersity Index (PdI) relates to the distribution. N/A: Not applicable (doses not included in the study)

#### Electron microscopic analysis of dispersed particles used for mouse instillation

**Fig. 2:**
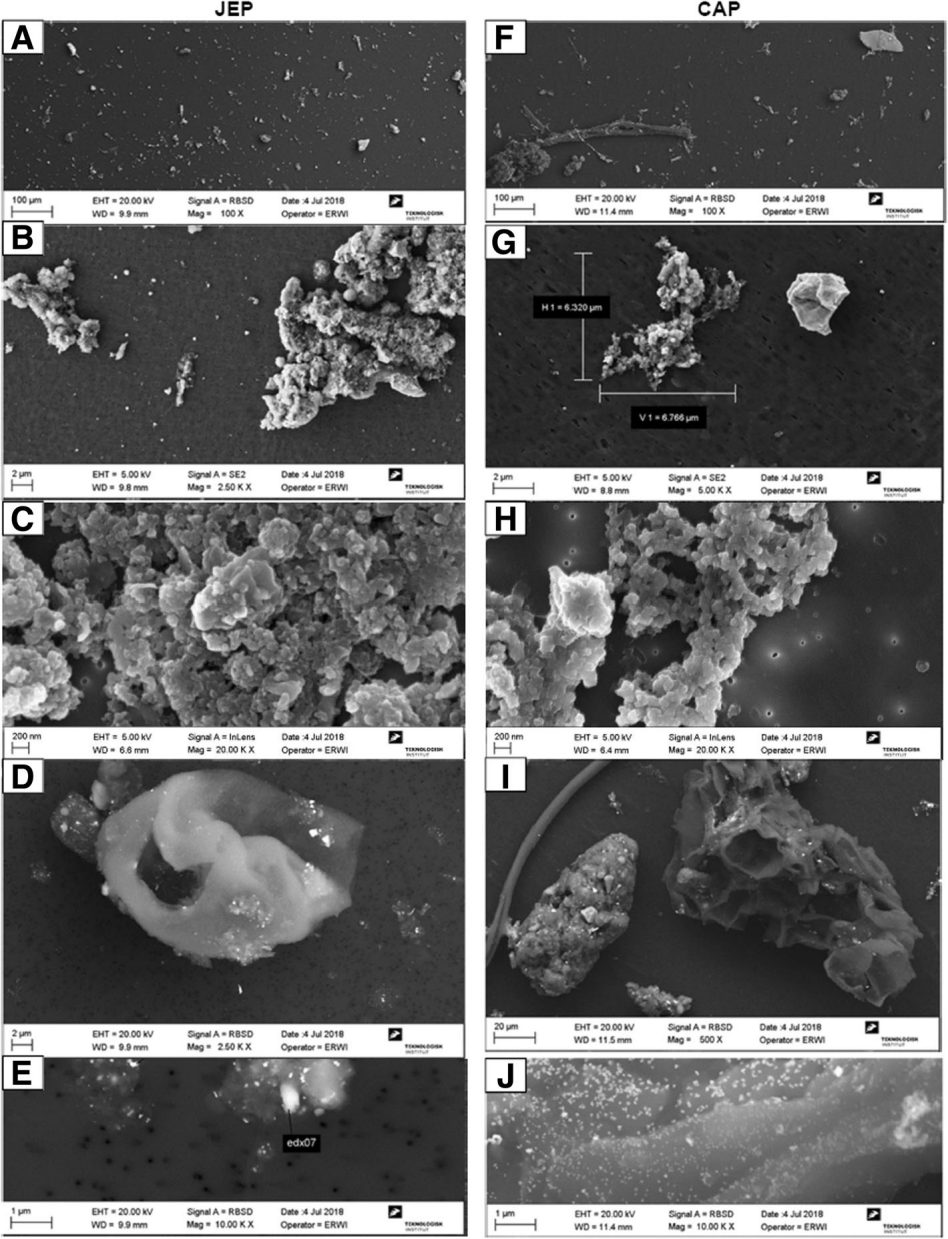
Scanning electron micrographs of collected particles dispersed in water. A + F: Overview of dispersed particles showing difference in homogeneity between JEP and CAP (bar: 100 μm). B + G: Detail of agglomerates consisting of smaller particles (bar: 2 μm). C + H: Detail of primary soot particles in agglomerates (bar: 200 nm). D + I: Details of collapsed pollen grains and plant fiber (bar: D; 2 μm, I; 20 μm). E + J: Details of silver particles covering agglomerates and plant fragments (bar: 1 μm)

In EM images, JEP appeared homogenous with small and larger aggregates and/or agglomerates of primary soot particles (Fig. 2a-c). A few organic structures, likely pollen, were also observed alongside large titanium particles (Fig. 2d and Additional file 1: Figure S1 E (1)), presumably originating from the titanium probe used for sonication. The estimated size of smaller particles forming larger JEP aggregates and/or agglomerates was approx. 45 nm. CAP appeared to be a more heterogenous mixture of particles (Fig. 2f-h) that also contained large plant fibers and collapsed pollen grains (Fig. 2i) along with smaller aggregates and/or agglomerates up to approx. 45 nm and silicates. In correspondence with results from the metal analysis, the EDS showed a heterogenic mixture of different metals and compounds, including silicon, titanium, iron, copper, magnesium, and zinc (Additional file 1: Figure S1 E (2)). The agglomerated soot particles, pollen and other organic elements of both JEP and CAP were decorated with silver (Ag) nanoparticles (Fig. 2e+j), which likely originates from the ESP silver plates. NIST2975 particles appeared as smooth-looking large carbon aggregates and/or agglomerates mixed with smaller fragments and clear metal reflections, consisting of mainly titanium. Silicon, iron and sulfur were also abundant. The large aggregates and/or agglomerates consisted of smaller similar-appearing particles or aggregates and/or agglomerates, of approx. 45 nm (Additional file 1: Figure S1 D (3)).

In summary, both JEP and CAP dispersions consisted of small-sized aggregated carbon particles, similar to standard diesel particles in size, shape, and chemical composition as measured by EDS. The JEP particles in suspension appeared homogenous compared to the CAP suspension and appeared to consist mainly of jet engine exhaust, whereas CAP suspension was more representative of the complex occupational exposure at the apron of the commercial airport.

### Pulmonary particle deposition and histopathology of exposed C57BL/6 mice

Female C57BL/6 mice were exposed to JEP, CAP, NIST2975, and CB by single intratracheal instillation at different dose levels and followed for 1, 28, or 90 days.

**Fig. 3:**
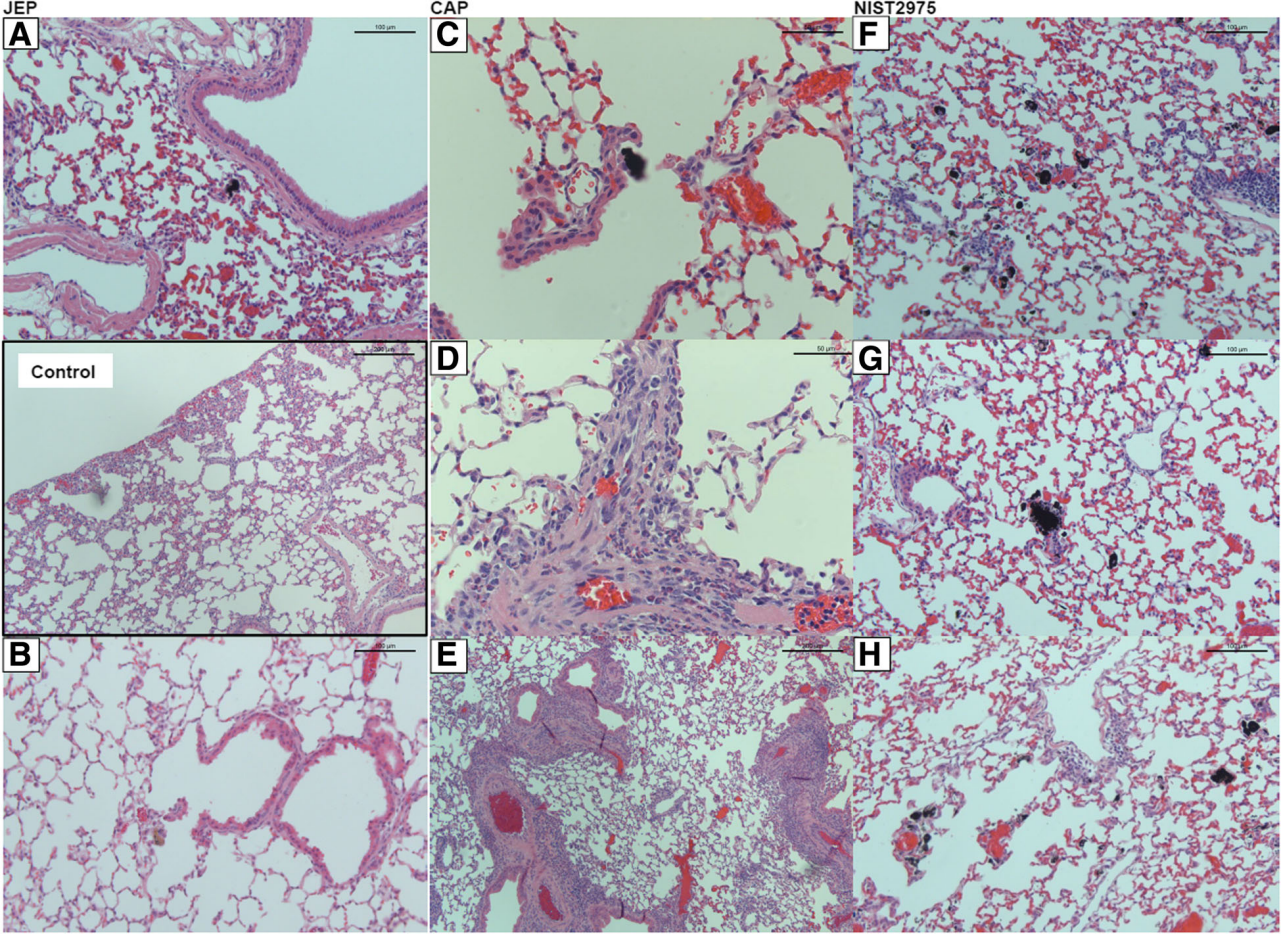
Histopathology of the lung on 28 and 90 days following exposure to 54 μg particles collected at a non-commercial airfield (JEP) and at the apron of a commercial airport (CAP). The sections were stained with HE. Control: Section of lung from a control mouse instilled with water only. A and B: Particles were not readily apparent in mice instilled with JEP and no significant pathological changes were found on day 28 or 90. C: In mice instilled with CAP, some particles were visible in macrophages. D and E: Pronounced eosinophil infiltration and eosinophil vasculitis was observed on day 28 and 90, characterized by infiltrates in the perivascular region and smooth muscle hyperplasia. F-H. Day 28 and 90. Lung sections of mice exposed to 162 μg NIST2975 had visible particles and particle-loaded macrophages, along with modest inflammation-related changes

Histopathological evaluation was performed on samples from mice exposed to 54 μg JEP, 54 μg CAP, and 162 μg NIST2975 on day 28 and day 90. The tissue samples showed heterogeneity between animals. Particles were not readily apparent in mice instilled with JEP particles and no significant histological changes were detected on day 28 and 90 (Fig. 3a+b).

In mice instilled with CAP, some particles were visible in macrophages (Fig. 3c) and on one occasion in a granuloma. Pronounced eosinophil infiltration and eosinophil vasculitis was observed on day 28, characterized by infiltrates in the perivascular region and smooth muscle hyperplasia (Fig. 3d+e). In the portal areas of the liver, eosinophilia was seen, most pronounced in mice exposed to CAP (not shown). This was also present in some JEP-instilled mice and in some control mice as well. Kidney and spleen were unaffected by exposure. NIST2975 lung sections had visible particles and particle-loaded macrophages (Fig. 3f-h), along with modest inflammation-related changes.

In summary, histological lung sections from day 28 and 90 post-exposure to airport particles showed small remnants of particles, likely due to clearance and relocation, and the pronounced degree of eosinophilic cell infiltrates especially in the CAP-instilled mice reflected the heterogenetic nature of CAP including pollen and plant fibers, which are associated with eosinophilic responses.

### BAL fluid cell composition

BAL fluid cellular content was evaluated by total cell count and composition of inflammatory cell subsets (Table 6, Additional file 2: Figure S2 A).

**Table 6:** BAL fluid cell composition on day 1, 28 and 90 post-exposure

	Total cell count	Neutrophils	Macrophages	Eosinophils	Lymphocytes	Epithelial cells
Day 1						
* Vehicle control*	56.43 ± 6.42	2.84 ± 0.89	46.58 ± 6.00	1.16 ± 0.79	0.84 ± 0.47	5.00 ± 1.59
* CB 54* μg	144.80 ± 16.23(****)	100.11 ± 11.85(****)	28.52 ± 3.05	9.96 ± 2.58(***)	1.43 ± 0.43	3.85 ± 0.78
* CAP 6 μg*	53.32 ± 9.87	6.43 ± 0.88	42.62 ± 8.90	0.30 ± 0.07	0.04 ± 0.04	3.94 ± 1.65
* CAP 18 μg*	82.22 ± 11.96	36.72 ± 10.00(***)(¤)	38.28 ± 2.94	1.40 ± 0.42	0.33 ± 0.15	5.49 ± 1.60
* CAP 54 μg*	147.50 ± 10.64(****) (¤¤¤¤)(“’)	101.09 ± 11.07(****) (¤¤¤¤)(“’)	38.79 ± 5.78	1.85 ± 0.58(*) (“)	0.67 ± 0.30	5.10 ± 1.24
* JEP 6* μg	66.37 ± 21.58	6.29 ± 3.00	43.83 ± 7.92	10.01 ± 9.69	1.46 ± 1.26	4.77 ± 1.40
* JEP 18* μg	91.02 ± 9.67	25.89 ± 8.57(*)	57.40 ± 3.43(xx)	1.58 ± 0.63	1.01 ± 0.38	5.15 ± 1.40
* JEP 54* μg	160.50 ± 17.40(****) (¤¤¤¤)(“’)	110.88 ± 14.66(****) (¤¤¤¤)(“’)	37.40 ± 6.94	4.27 ± 1.75(*) (“)	3.31 ± 1.05(¤) (“’)	4.65 ± 0.97
* NIST2975 18 μg*	47.92 ± 7.36	1.67 ± 0.46	42.05 ± 7.23	0.17 ± 0.09	0.19 ± 0.12	2.68 ± 0.51
* NIST2975 54 μg*	61.50 ± 9.22	25.57 ± 5.82(*)	31.28 ± 3.61	1.05 ± 0.41	0.93 ± 0.27	3.48 ± 0.75
* NIST2975 162 μg*	191.33 ± 11.98(****)	148.46 ± 9.74(****)	32.55 ± 4.13	5.07 ± 2.26	1.77 ± 0.59	4.78 ± 0.93
* NIST1650* ^*a*^ *18 μg*	87.55 ± 7.706	10.94 ± 2.78	62.91 ± 4.77	0.36 ± 0.13	0.09 ± 0.09	
* NIST1650* ^*a*^ *54 μg*	72.238 ± 8.993	12.07 ± 5.46	50.56 ± 3.23	1.03 ± 0.98	0.10 ± 0.07	
* NIST1650* ^*a*^ *162 μg*	177.375 ± 16.756	120.54 ± 11.80	48.92 ± 5.75	1.23 ± 0.49	0.21 ± 0.15	
Day 28						
* Vehicle control*	51.10 ± 3.89	0.10 ± 0.05	47.09 ± 3.89	0.16 ± 0.08	0.19 ± 0.08	3.55 ± 0.75
* CB 54 μg*	75.63 ± 13.40	1.68 ± 0.75(*)	66.83 ± 12.12	0.04 ± 0.04	3.20 ± 0.77(*)	3.88 ± 0.55
* CAP 6 μg*	50.20 ± 7.92	1.19 ± 0.84	42.50 ± 5.05	2.82 ± 2.64	1.03 ± 0.73	2.66 ± 0.88
* CAP 18 μg*	60.23 ± 8.02	1.05 ± 0.68	46.83 ± 2.83	7.90 ± 5.98	1.43 ± 0.80	3.02 ± 0.65
* CAP 54 μg*	48.85 ± 9.20	0.10 ± 0.07	39.18 ± 6.33	3.90 ± 2.76	2.42 ± 1.15(*)	3.25 ± 0.90
* JEP 6 μg*	51.47 ± 11.08	0.13 ± 0.08	49.07 ± 10.53	0.02 ± 0.02	0.26 ± 0.09	2.00 ± 0.64
* JEP 18 μg*	61.75 ± 7.01	0.27 ± 0.12	63.01 ± 4.84	0.23 ± 0.17	0.59 ± 0.25	2.94 ± 0.72
* JEP 54 μg*	46.43 ± 8.56	0.68 ± 0.38(*)	40.18 ± 7.73	0.14 ± 0.07	0.87 ± 0.37(*)	4.56 ± 1.37
* NIST2975 18 μg*	60.67 ± 7.71	0.27 ± 0.19	54.98 ± 7.61	0.09 ± 0.06	0.47 ± 0.11	4.87 ± 0.88
* NIST2975 54 μg*	50.37 ± 6.07	0.21 ± 0.18	43.35 ± 6.67	0.08 ± 0.05	0.45 ± 0.27	6.27 ± 1.88
* NIST2975 162 μg*	81.85 ± 8.40(*)	2.14 ± 0.92(**)	70.53 ± 7.43	0.30 ± 0.30	4.12 ± 1.93(**)	4.75 ± 0.43
* NIST1650*^*a*^ *18* μg	61.01 ± 3.13	0.44 ± 0.22	46.46 ± 2.98	0.11 ± 0.05	0.60 ± 0.12	
* NIST1650* ^*a*^ *54 μg*	58.26 ± 7.56	0.18 ± 0.07	45.83 ± 5.68	1.33 ± 0.78	1.27 ± 0.54	
* NIST1650* ^*a*^ *162 μg*	83.94 ± 10.64	1.86 ± 0.83	61.86 ± 6.29	0.28 ± 0.12	4.01 ± 1.25	
Day 90						
* Vehicle control*	54.03 ± 5.14	0.45 ± 0.16	45.23 ± 4.39	0.98 ± 0.91	3.57 ± 3.39	3.80 ± 0.74
* CB 54 μg*	86.93 ± 8.78(**)	2.07 ± 0.49(**)	73.33 ± 6.72	0.09 ± 0.09	4.70 ± 1.79	6.75 ± 1.58
* CAP 54 μg*	62.75 ± 4.30	0.92 ± 0.33	56.68 ± 4.52	0.10 ± 0.10	1.30 ± 0.82	3.74 ± 0.39
* JEP 54 μg*	50.90 ± 7.07	0.92 ± 0.47	42.69 ± 5.78	0.14 ± 0.07	1.74 ± 1.40	5.42 ± 1.51
* NIST2975 162 μg*	48.42 ± 7.09	0.46 ± 0.18	45.11 ± 6.94	0.00 ± 0.00	0.38 ± 0.21	2.47 ± 0.44

^a^NIST1650 data was included for comparison and obtained from a previously published study (Kyjovska et al. Mutagenesis 2015)

*P*-value summary: (*) – (****) = *p* < 0.05 - *p* < 0.0001 increase compared to vehicle control, (x) – (xxxx) = *p* < 0.05 - *p* < 0.0001 increase compared to CB 54 μg, (¤) – (¤¤¤¤) = *p* < 0.05 - *p* < 0.0001 increase compared to NIST2975 of same dose, (‘) – (““) = *p* < 0.05 - *p* < 0.0001 increase compared to NIST1650 of same dose. Data are shown as Mean ± SEM (× 10^3^)

*BAL* broncho-alveloar lavage, *CAP* commercial airport particles, *JEP* jet engine particles, *CB* carbon black Printex 90

#### BAL cells on day 1 post-exposure

**Fig. 4:**
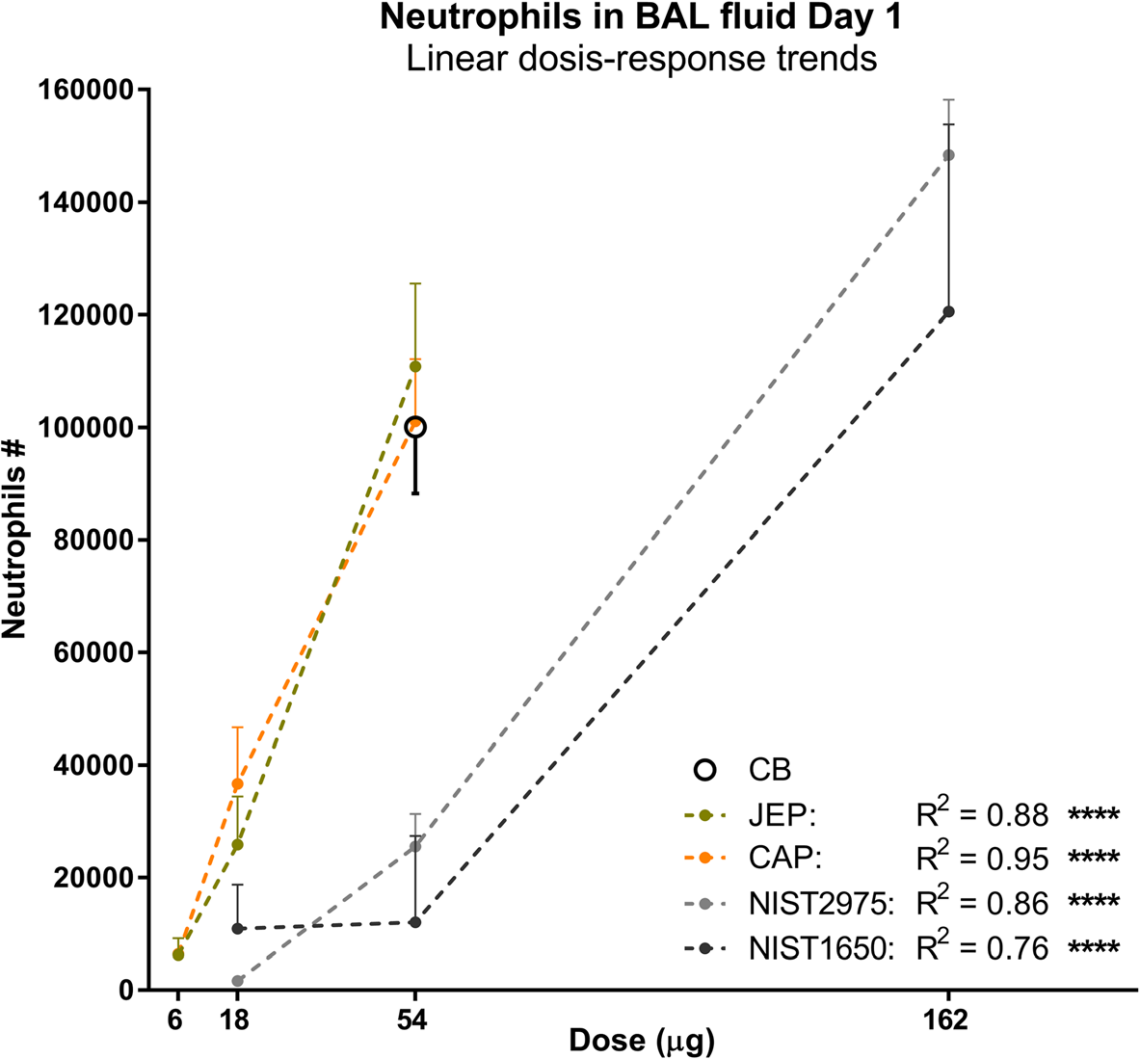
Illustration of dose-response linearity between instilled doses of airport-collected particles, NIST2975, NIST1650 and neutrophil influx in BAL. Increasing dose-response effects were confirmed with test for linear trend, where the *alerting R2* (referred to as R2) is the fraction of the variance between group means that is accounted for by the linear trend (Altman/Sheskin, provided by GraphPad Prism). Data for NIST1650 was obtained from a previously published study [19]. Significant linear trends were verified for total cell numbers (not shown) and neutrophils in BAL fluid, with R2 between 0.76 and 0.95

On day 1 post-exposure, dose-response relationships were observed for JEP, CAP, and NIST2975 for total cell count, neutrophils, eosinophils, and lymphocytes. Significant linear trends were verified for the observed dose-response relationships for neutrophils and total cell numbers (not shown) with R-square values between 0.76 and 0.95 (Fig. 4).

**Fig. 5:**
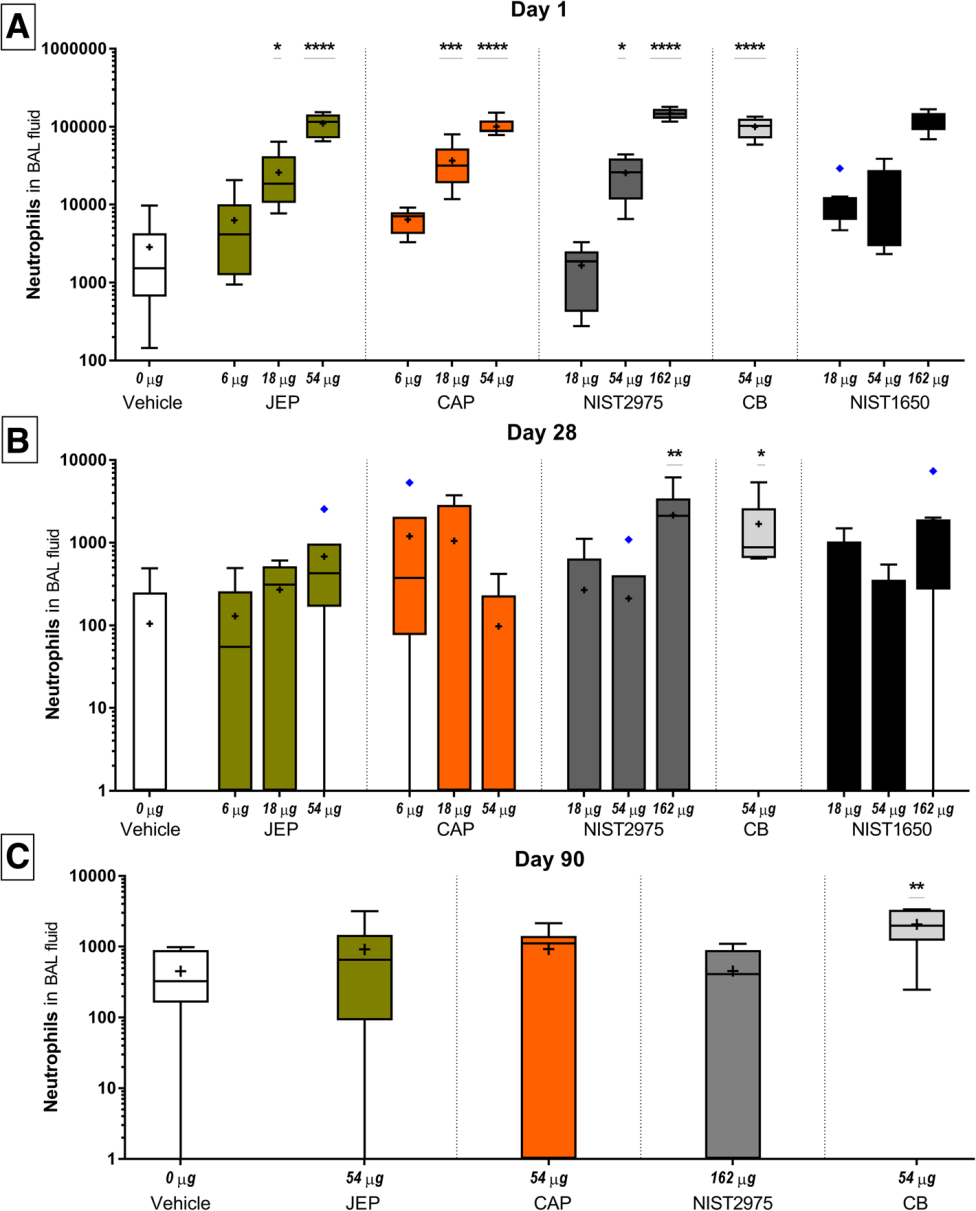
Neutrophil influx in BAL fluid on day 1, 28, and 90 following exposure to jet engine particles (JEP), commercial airport particles (CAP), and reference particles NIST2975, NIST1650, and Carbon black Printex90 (CB) (Tukey plots, +: mean, line: median, diamonds: outliers). Mice were exposed to 6, 18, and 54 μg of JEP and CAP, to 54 μg of CB, and to 18, 54, and 162 μg of NIST particles with 6 mice in each group. Data for NIST1650 was obtained from a previously published study [19]

Exposure to JEP and CAP at 18 and 54 μg resulted in significantly increased neutrophil influx, compared to vehicle control (JEP 18 μg: *p* = 0.0215, JEP 54 μg: *p* < 0.0001, CAP 18 μg: *p* = 0.0008; CAP 54 μg: *p* = 0.0001) (Fig. 5a). In addition, at 54 μg, JEP- and CAP-exposure induced significant eosinophil influx, compared to vehicle control (JEP: *p* = 0.0158, CAP 54 μg: *p* = 0.0205) (Additional file 2: Figure S2A (4)). By exclusion of statistically determined outliers (see In vivo data statistics), this difference was further increased (JEP: *p* = 0.0011, CAP: *p* = 0.001) with an addition of significance for 18 μg as well (JEP: *p* = 0.0422, CAP: *p* = 0.0139). By removal of outliers in lymphocyte counts, there was an additional significant difference between JEP at 54 μg and vehicle control (*p* = 0.0004) (see Additional file 2: Figure S2 A (1)). However, the results were qualitatively similar with and without outliers.

Exposure to 54 μg CB significantly increased total cells (*p* < 0.0001), neutrophils (*p* < 0.0001) and eosinophils (*p* = 0.0002) compared to vehicle controls. NIST2975 instilled mice had significantly increased cell numbers compared to vehicle for neutrophils at 54 μg (*p* = 0.0299) and at 162 μg (*p* < 0.0001) (Fig. 5a). It was apparent that CB 54 μg, NIST 162 μg, and the two airport-collected particles JEP and CAP at 54 μg induced similar responses when compared to vehicle control for most of the assessed cell types, and that JEP and CAP responses were increased when compared to same mass dose of NIST2975 and NIST1650b (Table 6). There was the expected dose-response relationships between total deposited surface area for CB (182 m^2^/g for CB [33]), NIST2975 (91 m^2^/g) [15], NIST1650 (108 m^2^/g) [15] and neutrophil influx (Additional file 2: Figure S2A (4)).

#### BAL cells on day 28 post-exposure

On day 28 post-exposure there was still a significant increase in neutrophil numbers compared to vehicle controls for JEP at 54 μg (Fig. 5b), and a significantly increased number of lymphocytes for both JEP and CAP at 54 μg dose level (JEP: *p* = 0.0328, CAP: *p* = 0.0223). Total cell count for NIST2975 162 μg were still significantly increased compared to vehicle control (*p* = 0.0153) (Table 6). Neutrophil counts for CB and NIST2975 at 162 μg were still significantly increased compared to vehicle control (CB: *p* = 0.446; NIST2975: *p* = 0.0068) (Fig. 5b). In addition, there was a significant increase for lymphocytes (CB: *p* = 0.0228; NIST2975 162 μg: *p* = 0.0023) (Table 6). By removing statistically determined outliers, this difference was increased (CB: *p* = 0.0001; NIST2975: *p* = 0.0031).

#### BAL cell on day 90 post-exposure

Mice from the highest dose groups were followed until day 90 post-exposure, and there was still increased total cell counts (*p* = 0.0022) and neutrophils (0.0045) for CB, compared to vehicle control mice (Fig. 5c, Table 6, and Additional file 2: Figure S2 A (3)).

In summary, both JEP and CAP particles induced high pulmonary inflammatory responses on day 1 post-exposure, similar or higher compared to same mass dose of NIST control particles and CB. On day 28, there was still active inflammation in mice exposed to JEP and CB, and CB still induced increased neutrophil influx on day 90.

### Serum amyloid a

Serum amyloid (Saa) 3 (*Saa3*) mRNA in lung tissue and *Saa1* mRNA levels in liver tissue were used as biomarkers of pulmonary [34] and hepatic [35] acute phase response, respectively. SAA3 protein was measured in plasma as biomarker of systemic acute phase response [35]. *Saa* expression in lung and liver was measured on day 1, 28 and 90 post-exposure, and SAA3 in plasma on day 1 and on day 28 for the highest particle doses.

**Fig. 6:**
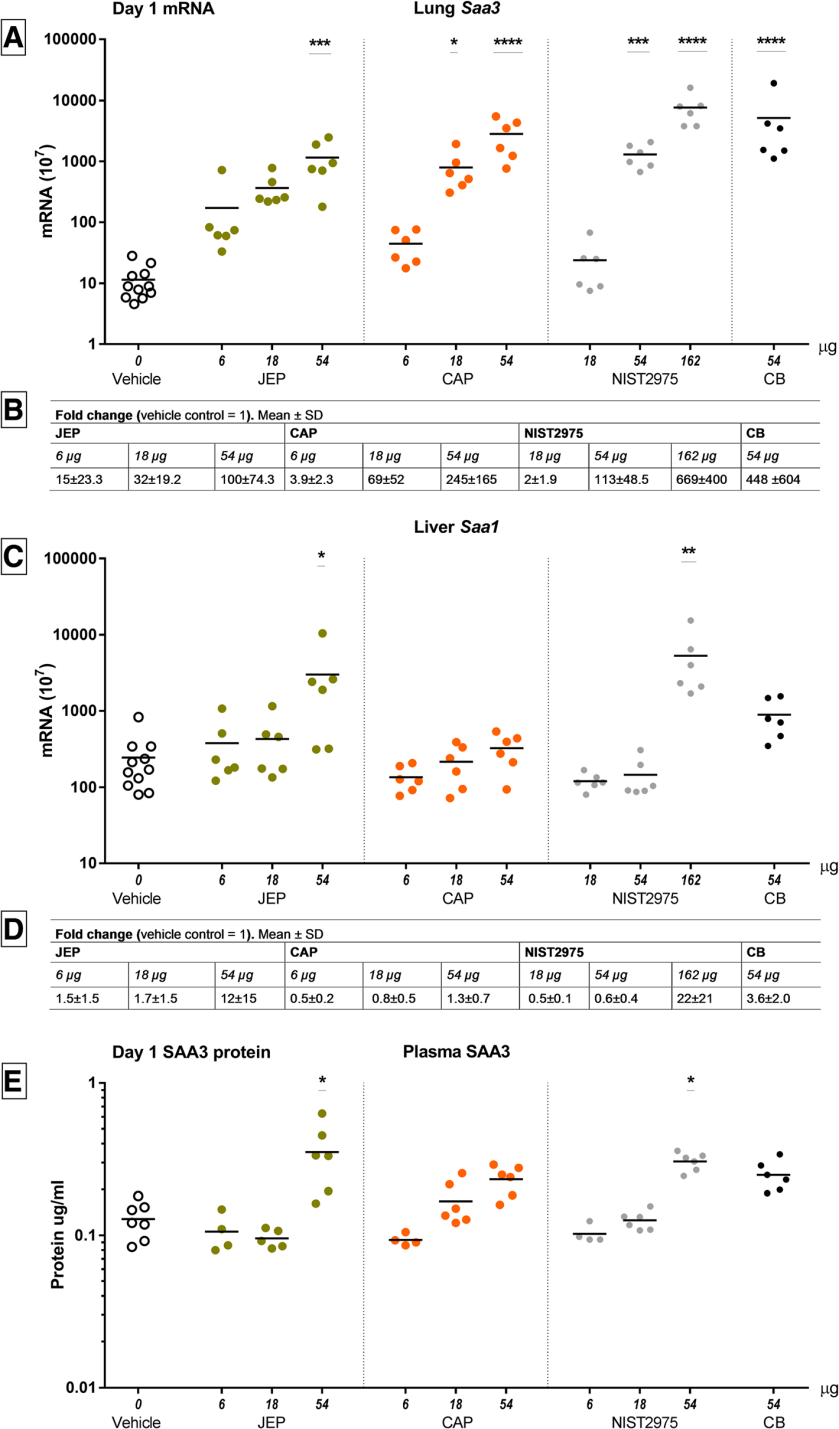
mRNA levels of *Saa3* in lung, *Saa1* liver, and SAA3 plasma protein on day 1 (scatter plots, mean + SEM). *Saa3* mRNA in lung tissue and *Saa1* mRNA in liver tissue were used as biomarkers of pulmonary and hepatic acute phase response, following exposure to particles collected at the apron of a commercial airport and in a jet shelter at a non-commercial airfield. SAA3 protein was measured in plasma. *Saa* in lung and liver was measured on day 1, 28 and 90 post-exposure, and SAA3 on day 1 and on day 28 for highest particle doses

Exposure to JEP, CAP and NIST2975 resulted in significant dose-dependent increases in *Saa3* mRNA levels in lung tissue compared to vehicle control mice on day 1 (CAP 18 μg: *p* = 0.0151, CAP 54 μg: *p* = < 0.0001, JEP 54 μg: *p* = 0.0038, NIST2975 54 μg: *p* = 0.0008, NIST2975 162 μg: *p* < 0.0001) (Fig. 6a+B). CB induced a 447-fold *Saa3* mRNA level increase (*p* < 0.0001) (Fig. 6), in agreement with previous findings [36]. On day 90, *Saa3* mRNA levels in the CB-exposed group were still increased compared to control (day 90: *p* = 0.0192) (Additional file 2: Figure S2 B). On day 1, liver *Saa1* mRNA levels were significantly increased for JEP of 54 μg, compared to control (*p* = 0.0415; 12-fold increase) and for NIST2975 of 162 μg (*p* = 0.0025, 22-fold increase) (Fig. 6c and d). On day 1 post-exposure, plasma SAA3 was increased for JEP 54 μg (*p* = 0.0305) and for NIST2975 at 162 μg (*p* = 0.0205) (Fig. 6e). No significant differences were found for *Saa1* mRNA in liver tissue or for SAA3 plasma protein level on day 28 (see Additional file 2: Figure S2 B).

Thus, JEP and CAP exposure induced dose-dependent pulmonary acute phase response on day 1 post-exposure that was paralleled by a systemic circulation of SAA3 protein for JEP. The acute phase response had returned to baseline levels on 28 days post-exposure for JEP, CAP, and NIST2975.

### DNA damage

**Fig. 7:**
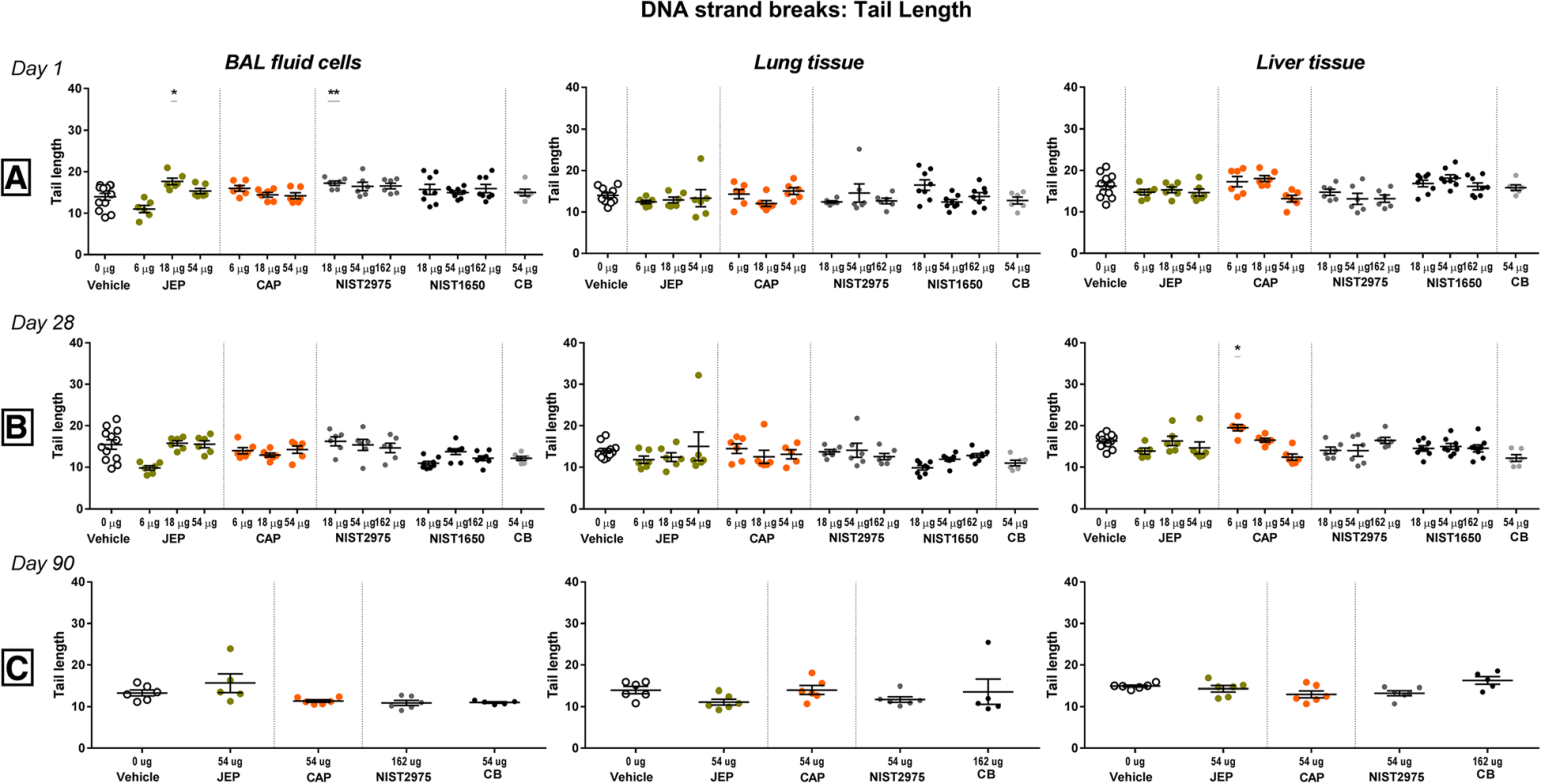
DNA strands break levels evaluated by tail length in the Comet assay on day 1, 28, and 90 following exposure to jet engine particles (JEP), commercial airport particles (CAP), and reference particles NIST2975, NIST1650, and carbon black Printex90 (CB) (scatter plots, mean + SEM). Mice were exposed to 6, 18, and 54 μg of JEP and CAP, to 54 μg of CB, and to 18, 54, and 162 μg of NIST particles. Data for NIST1650 was obtained from a previously published study [19]

Genotoxicity was evaluated as DNA strand breaks in the comet assay, using comet tail length and % tail DNA in BAL derived cells, lung cells and liver cells. Increased levels of DNA strand breaks were occasionally observed across particles types, dose and time points, but no dose-response relationships was observed (Fig. 7 and Additional file 2 S2 C).

#### DNA damage on day 1 post-exposure

On day 1 post-exposure, increased DNA damage levels were observed for JEP and NIST 2975 at 18 μg as compared to vehicle control (JEP: *p* = 0.0132, NIST2975: *p* = 0.0304) for tail length in BAL cells (Fig. 7a and Additional file 2 S2 C).

#### DNA damage on day 28 post-exposure

On day 28, tail length and % tail DNA (see Additional file 2 S2 C) in liver cells were increased compared to vehicle control for CAP 6 μg (% tail DNA: *p* = 0.0151; tail length: *p* = 0.0214) (Additional file 2 S2 C and Fig. 7b).

#### DNA damage on day 90 post-exposure

On day 90, there were no significant differences compared to vehicle controls (Fig. 7c and Additional file 2 S2 C).

In summary, increased levels of DNA strand breaks were observed in single dose groups on day 1 and 28 post-exposure, with a pattern of most DNA damage in BAL cells for JEP and in liver cells for CAP.

## Discussion

In this study, mice were exposed to particles collected at two different airport facilities and compared to standard diesel particle NIST2975 and to published data on NIST1650. With ESP collection, 11.7 mg of JEP were collected during a time span of approximately 15 h and 12.3 mg particles of CAP were collected at the commercial airport during 4 h and 40 min. JEP and CAP both contained metals and PAH. Total PAH content was similar to the declared content of NIST2975 and substantially lower than for NIST1650. The metal contents in the CAP and JEP were considerably higher than for NIST2975.

The sizes, shapes and structures of the primary soot particles found predominantly in JEP and also in CAP samples were very similar to those found in NIST2975 and to particles from previous studies [37]. Thus, they likely have comparable surface area and physicochemical properties.

### Inflammation

After intratracheal instillation in mice, both JEP and CAP particles produced highly increased influx of inflammatory cells in BAL fluid on day 1 post-exposure, similar or higher compared to same mass dose of NIST control particles and CB. On day 28, there was still influx of inflammatory cells in BAL fluid in mice exposed to JEP and CB. Only CB still induced increased cellular responses on day 90. We used water as vehicle for intratracheal instillation to ensure least amount of vehicle-induced artefacts [32]. The inflammatory profile on day 1 post-exposure could potentially be partly attributed to lipopolysaccharides (LPS) from the air and environment, however, the inflammation was still present on day 28 post-exposure, which would not be expected from acute inflammation mediated by organic material. As an example of the pulmonary response to organic material, inflammation induced by pulmonary exposure to bulk cellulose was observed 1 day post-exposure, but not 28 days post-exposure [38]. The histopathological evaluation of lung tissue showed limited JEP and CAP-inflammatory changes 28 and 90 days post-exposure.

We did not collect sufficient material to determine BET surface area, and therefore, we could not compare the inflammatory response induced by JEP and CAP with standard diesel particles and CB-induced inflammation when normalized to surface area. However, we observed strong mass dose-dependency. The cytological changes in BAL fluid induced by CAP and JEP were remarkably similar. Assuming that the combustion particles indeed have a diameter of 10 nm as our data suggested, then the specific surface area of JEP and CAP would be at least similar to that of CB, which has a diameter of 14 nm and BET of 182 m^2^/g [33]. The BET of NIST1650 and 2975 are 108 m^2^/g and 91 m^2^/g, respectively. We found dose-response relationships between total deposited surface area for CB, NIST2975, NIST1650 and neutrophil influx. Thus, the observed stronger inflammatory response, as determined by BAL, induced by JEP and CAP compared to NIST2975 would be consistent with the expected larger specific surface area of the smaller jet engine combustion particles.

### Acute phase response

*Saa3* mRNA levels were used as biomarker of pulmonary acute phase response [34]. Particle-induced dose-dependent pulmonary acute phase response was observed in parallel with the neutrophil influx as previously reported for CB and NIST1650b [23, 34]. The hepatic acute phase response evaluated with *Saa1* mRNA levels was much smaller than the pulmonary acute phase response, as previously seen for NIST2975 and CB [36, 39]. Systemic SAA3 levels were also increased by JEP exposure at 54 μg, and by NIST2975 at the three fold higher dose 162 μg. SAA is causally related to increased plaque progression [40] and SAA stimulates the formation of macrophages into foam cells [41]. Increased levels of acute phase proteins SAA and C-reactive protein (CRP) are associated with increased risk of cardiovascular disease in prospective epidemiological studies [42]. Furthermore, inhalation of ZnO nanoparticles increased systemic levels of CRP and SAA in human volunteers in a dose-dependent manner [43].

### Genotoxicity

Increased levels of DNA strand breaks were observed with the Comet assay at single dose levels across doses and post-exposure time points, with a pattern of most DNA damage in BAL cells for JEP and in liver cells for CAP. BAL cells are not relevant cell types in relation to lung cancer, but may be more homogeneously exposed to particles following IT exposure as compared to epithelial cells, even though we have previously documented that IT exposure result in exposure of all lung lobes [2, 44]. The observed levels of DNA damage were overall low, but at the same level as for the NIST diesel particles and CB [23]. We have previously validated our comet assay set up for in vivo samples using chemical-induced DNA damage and found strong dose-response relationships in all assessed tissues [45]. We have previously assessed DNA damage in BAL cells, lung and liver tissue of mice after pulmonary exposure to many different nanomaterials [23, 25, 44, 46–50]. As previously discussed [23], we observe the same lack of dose-response relationship in the three tissues in the majority of our studies. Instead of dose-response relationship, we generally observe that particle exposure at all dose levels increases the level of DNA strand breaks with 50–100%, an increase that will only be statistically significant in some cases depending on the variation in the assay. The lack of dose-response relationship may indicate a maximal rate of particle-induced DNA strand breaks was achieved already at low doses. This, in turn, could indicate that particle-induced DNA strand breaks in the lung are formed by a mechanism that is fundamentally different from chemically-induced DNA damage [23]. CAP exposure induced DNA strand breaks in liver tissue, as previously observed for CB [36, 46]. We have recently shown the genotoxicity in liver following pulmonary exposure to CB is likely caused by direct genotoxicity caused by surface-dependent reactive-oxygen-species (ROS) generation of translocated particles [51]. Translocation from lung to systemic circulation is very size-dependent, and consistent with this, the primary airport-collected combustion particles were small (10–30 nm in diameter).

### Metals and PAH

Both CAP and JEP contained toxic metals including lead, cobalt, nickel, arsenic, cadmium and mercury, measured with ICP-MS. The content of Ag in JEP and CAP was likely attributed to contaminations from the ESP silver plates. Our analysis of the reference particles NIST2975 and CB were in overall agreement with the literature [24]. The discrepancy between the current study and previously published values for NIST2975 [24, 25] may be caused by longer extraction times and the use of 25% nitric acid instead of phosphate buffer.

In our study, the ∑PAH concentration was 0.081 mg/g in CAP and 0.05 mg/g in JEP, respectively. In comparison, CB was previously shown to contain 0.000074 mg/g PAH [52], NIST2975 contains 0.086 mg/g and NIST1650b contains 0.22 mg/g of the 16 PAH according to NIST [15, 23]. However, based on 2-year inhalation studies in rats, it was previously concluded that the carcinogenic effect of diesel exhaust particles cannot be explained by the content of carcinogenic PAH alone [12, 53]. Likewise, inhalation of carbon black nanoparticles was just as carcinogenic as diesel exhaust in a 2 year inhalation study in rats, suggesting that the carbon core of the particles contributes significantly to the carcinogenic effect of diesel particles [14]. In vitro, NIST1650 and Printex 90 carbon black nanoparticles had similar mutagenic potential in the murine fibroblast cell line FE-1 [52, 54]. Thus, even though CAP and JEP have similar PAH content as NIST2975, the carbon particle core is likely an important driver of pulmonary toxicity as previously observed for diesel particles and carbon black nanoparticles.

### Histology and doses

JEP and CAP appeared different on EM images. CAP induced a higher eosinophil response compared to JEP, reflecting the complex mixture of the commercial airport air with pollen and plant fibers, compared to the more homogenous jet engine sample. Histological examination of lung and liver tissue revealed eosinophilic pulmonary vasculitis in CAP-exposed mice, likely reflecting the exposure to pollen grains, which can be associated with allergic response. This type of histopathology was previously reported in association with asthma models in mice [55]. To the best of our knowledge this has previously not been reported in association with particle exposures. The samples for histology were collected on day 28 and 90, and generally very few particle agglomerates were observed in 54 μg JEP- and CAP-exposed mice, in contrast to mice exposed to the 3-fold higher dose of 164 μg NIST2975 reference particles. The smallest retained dose seemed to be in JEP-exposed mice, where in most cases no material could be detected. This could be due to clearance of particles from lungs and liver before day 28, or because the JEP de-agglomerated in the lung and single JEP were too small for detection by conventional microscopy.

### Distribution and human risk

Environmental ESP particle collection, extraction, dispersion, and instillation are all experimental procedures that may modify the final deposited material in mice lungs as compared to occupational inhalation exposure. The impactor EM images represent the mixed ambient air contents, but are not necessarily a representative sample of aerosol contents over time, as the impactor efficiency varies with particle size and sample collection time was short. The ESP collection method seems to have contributed with additional silver (Ag) to the CAP and JEP suspensions instilled in the mice, which was not present in the reference particles. However, the silver mass content was very low. Titanium nanoparticles were also detected, likely originating from the sonication probe. The vehicle control was also sonicated to account for sonication bias. High amounts of sea salt crystals were apparent in the impactor sampling of CAP, reflecting close proximity to the sea. This might result in higher particle CAP aerosol measurements. These salt crystals were absent in EM images of particles in suspension, since the salt dissolves in the water used as vehicle. JEP appeared to have low background levels, based on the low number densities on the impactor grids representing background exposure. JEP impactor samples were in turn dominated by soot particles, representing collection in the proximity of a running jet engine during taxi.

Occupational exposure tracking of JEP showed that the main combustion events of the jetfighter (plane leaving and plane arriving) resulted in high exposure levels, including in the breathing zone monitor of the airfield personnel. The average exposures and doses of one full cycle of 170 min were measured to yield at least 4.12 × 10^12^ particles, where 9.6% were predicted to deposit in the alveolar region of the lung. A comparison of all the DiSCmini event peaks (including breathing zone) suggested that the shelter room air volume is continuously mixed and that the actual geometrical measuring point is of less importance. Both the turbofan taking in large quantities of air and the airflow exiting the jet engine nozzle are sufficient to drive the jet shelter ventilation. There was a larger variation in DiSCmini signals in later stages of the second jetfighter occupational cycle, which can be attributed to local activity in the sampling volume and to instrument drift after extensive measuring time. In the current study, event-dependent air concentrations of up to 1000 μg/m^3^ were measured. Based on the size distribution data in the exposure measurements and assuming 1.8 L/h ventilation for mice [56], the estimated alveolar deposited dose for a mouse at 1000 μg/m^3^ for an 8-h workday would be:

(1000 μg/m^3^) x (8 h) x (1.8 L/h) × 0.096 = 1.38 μg deposited material/8 h workday.

The mice were instilled with the collected particles at doses 6, 18 and 54 μg. We therefore estimate that the lowest dose of 6 μg and the highest dose of 54 μg JEP and CAP in this study equals to 4 and 39 workdays, respectively.

The physicochemical characterization of JEP suggests that JEP are comparable to the standard diesel particles and carbon black Printex 90 (CB in this study). The inflammatory and genotoxic responses following pulmonary exposure to JEP were similar to standard diesel particles and CB. The biological response following pulmonary exposure to CAP was very similar to JEP even though CAP appeared more heterogeneous on EM images. This was seen as pronounced eosinophilic cell infiltrates in CAP-instilled mice, reflecting the contents of organic material including pollen and plant fibers, which are associated with eosinophilic responses.

In a recent meta-analysis of the association between occupational exposure to diesel exhaust and lung cancer, it was estimated that occupational exposure to 1 μg/m^3^ diesel exhaust particles measured as elemental carbon would induce 17 excess lung cancer cases per 10,000 exposed humans [11]. This warrants continuous research in reduction of particle emissions and diesel engine refinements, to ensure more efficient combustion to reduce particles both in diesel-origin emissions and in jet engines. Given the results in this study and further resemblance between JEP and diesel exhaust particles as well as the dose-response relationship between diesel exhaust exposure and lung cancer, the observed occupational exposure to jet engine emissions at the two airfields should be minimized.

## Conclusions

In conclusion, we collected particulate matter from the ambient air at two different airport facilities, a non-commercial airfield (JEP) and a commercial airport (CAP). The physicochemical characterization showed that JEP were primarily agglomerated carbon nanoparticles with levels of metals and PAH comparable to those found in the standard diesel particles NIST2975 and NIST1650. CAP was more heterogeneous and contained large organic particles, agglomerated carbon nanoparticles and condensed volatile organic compounds and was representative of the complex occupational exposure on the apron of a commercial airport. Pulmonary exposure to JEP and CAP induced acute phase responses as well as time and dose-dependent cytological changes in BAL cell composition, which were similar to the responses observed for NIST2975 and CB, and to previously published results for NIST1650. JEP, CAP and NIST2975 induced increased levels of DNA strand breaks across doses and time points. Our study suggests that jet engine particles have similar physicochemical properties and toxicity as diesel exhaust particles.

## Methods

### Particle collection, characterization and preparation

See Table 1 for an overview of measurements and instruments.

#### Non-commercial airfield particle exposure measurements.

Sampling stations were placed in a jet shelter of 4721 m^3^ (see Additional file 1: Figure S1 A) to measure the airborne particle concentrations in the near field, far field and in the breathing zone of the flight personnel. In order to track occupational exposure, two full cycles representative of a normal workflow were observed of Plane Leaving (PL), Plane Arriving (PA) and refueling by a Fuel Truck (FT).

#### Measurement strategy

Real-time particle monitoring was performed with an Electrical Low Pressure Impactor (ELPI, Dekati model ELPI+, Dekati Ltd., Tampere, Finland) and four DiSCmini (Matter Aerosol AG, Wohlen, Switzerland) deployed at several locations – ELPI at position 1 and DiSCmini at positions 2–4 and P (personal breathing zone) (Additional file 1: Figure S1 A). DiSCmini is a compact and portable instrument that measures particle number concentration, mean particle size and lung-deposited surface area (LDSA) [57]. LDSA is correspond to lung deposited surface area of particles in size range of ca. 20 to 400 nm for males during light exercise [58]. This method has high uncertainties, which are discussed in details by Koivisto et al. [59]. ELPI collects and classifies particles in a cascade impactor system according to aerodynamic mobility [60]. By combining these two instruments airborne particles with diameters from approx. 6 nm to 10 μm can be characterized with a detection emphasis on nanoparticles (DiSCmini optimum range is 10–700 nm) and particle concentrations up to 10^6^ particles/cm^3^ for DiSCmini and up to 10^8^ particles/cm^3^ for the ELPI.

#### Particle number to mass conversion

Particle number size distributions measured by the ELPI were converted to mass distributions by assuming that particles effective density is equal to nonvolatile effective particle density measured from a CFM56-5B4/2P turbine engine [61]. The size dependent relation given by Johnson et al. [55] for CFM56-5B4/2P turbine engine is

$$ {\rho}_{eff}=11.92\times {d}_p^{\left(2.76-3\right)}\ \left[\mathrm{kg}\;\mathrm{m}-3\right] $$

The respirable mass distribution (mPM4) was calculated by multiplying the particle mass size distribution by the simplified respirable fraction penetration efficiency according to Hinds [62].

#### Calculating deposited dose of inhaled particles

Particle deposition rates were calculated from particle concentrations measured by the ELPI. Particle concentrations were multiplied with the simplified ICRP [63] human respiratory tract deposition probabilities for the upper airways, the tracheobronchial region, and the alveolar region [62]. The respiratory minute volume was assumed to be 25 L/min, which corresponds to the typical respiration rate of a 70 kg male during light exercise (dose rates are described in detail elsewhere [64]).

#### Commercial airport measurements

Particle concentrations were measured using four DiSCminis and a NanoScan (TSI NanoScan model 3091, TSI Inc., Shoreview, MN, USA) for particles from 10 to 420 nm in 60 s intervals (Additional file 1: Figure S1 A).

#### Impactor collection

Aerosol samples were collected at a non-commercial airfield and commercial airport using a three stage cascade impactor, referred to as the Micro INertial Impactor or MINI [65]. A diaphragm gas pump model NMP 830 (KNF Neuberger, Germany) was used to generate the flow through the MINI, resulting in a flow rate of 0.76 L/min. At ambient conditions this gives theoretical cut-off diameters of 1.36, 0.59, and 0.055 μm [66]. Each stage of the MINI can be equipped with TEM grids, allowing particle collection directly onto microscope-suited surfaces. Here the stages were equipped with 400 mesh nickel TEM grids coated with a 10 nm Formvar substrate with 1 nm carbon deposited on top (Electron Microscopy Sciences, USA). Nickel grids were chosen as they are magnetic, thereby allowing them to be held in place with weak magnets, which were inserted into the impactor stages from the bottom. This ensured minimal movement of the grids during sampling.

#### Particle collection for physical and chemical characterization and mouse instillations

Respirable dust (PM4; particles below 4 μm in diameter, see definition from the European Committee for Standardization [67]) was collected using three sampling cyclones (BGI Model GK2.69, BGI Inc., Waltham, MA, USA) at volume flow of 4.2 L/min on 37 mm PTFE filters with a 0.8 μm pore size (Millipore, Billerica, MA, USA). The collections were 1) with a running jet engine, and 2) when there was no jet engines on in close vicinity, and 3) sampled over the measurement day. Particles for suspensions were collected by a commercial electrostatic precipitator (ESP) without using a prefilter, originally characterized by Sharma et al. [24], and previously used for sampling in a range of particle exposure studies [25, 26]. The collected particles were freeze dried for further processing.

#### Electron microscopy

The particles were visualized and characterized by electron microscopy, both from direct impactor collection and in suspension following ESP collection.

The impactor samples were analyzed with a Nova NanoSEM 600 (FEI, The Netherlands), equipped with an OPTIMUS TKD detector (Bruker, Germany), functioning as a scanning transmission electron microscopy (STEM) detector. The SEM was operated in high vacuum mode with acceleration voltages of 10–20 keV, a probe current of 12 nA, and at magnifications varying between 5 k and 40 k, corresponding to resolutions of 15 to 2 nm/pixel respectively. The Esprit software (Bruker, Germany) was used for automated analysis of the samples, where an imaging pattern was defined to cover an entire square of the TEM grid. The square chosen for analysis was situated directly under the impactor orifice and therefore displayed a high particle number density. Once the imaging routine is setup the software automatically acquire the images, segments them using a mean adaptive threshold technique, and performs subsequent energy dispersive x-ray (EDS) analysis on recognized particles larger than a given size criteria. For these samples the minimum particle size accepted for EDS analysis was set to 200 nm, as smaller particles were found to give limited x-ray counts. Exposure times for the EDS analysis was set to 30 s. Particles touching the image borders were discarded, as well as particles with equivalent circular diameters (ECD) smaller than 50 nm. The size criteria were necessary to minimize the number of misclassified substrate artefacts, which sometimes occurred during the automated analysis.

The impactor samples from the lowest stage were also analyzed at higher magnification using a Tecnai T20 G2 (FEI, Netherlands) TEM microscope. The TEM was operated in high vacuum mode, at an acceleration voltage of 200 keV, and with a probe current of 38 nA. In the TEM resolutions up to 0.02 nm/pixel were achieved, allowing visualization of the onion like structure of collected soot particles. In order to determine primary particle sizes of agglomerates the TEM images were analyzed manually with the open source image analysis program ImageJ (https://imagej.net/Citing). Particles in suspension were analyzed by field emission scanning electron microscopy SEM-EDX (ULTRA-55, Carl Zeiss NTS GmbH, Oberkochen, Germany) equipped with an energy dispersive X-ray spectroscopy system (Oxford X-Max 50 mm2, Oxford Instruments, Oxfordshire, UK). The particles were filtered onto Nucleopore Membranes with a hole size of 0.1 μm and hereafter carbon-coated by carbon thread evaporation. SEM images were acquired at magnifications between 100 and 50.000X and high tension at 5 and 20 kV. Detectors used were SE2, InLens and RBSD for options of visualizing surface topology, high resolution details, or material contrast. Identification of elemental composition identification was carried out with x-ray spectra acquired at 20 kV with a live time of 30 s.

#### Positive control particles

Carbon black Printex90 used as benchmark particle with previously well-characterized properties [16, 52, 68] was provided by Evonik Degussa GmbH (Frankfurt, Germany). Benchmark diesel particle SRM 2975 (referred to as NIST2975) was obtained from the National Institute of Standards and Technology (Gaithersburg, MD, USA). The certificate of analysis is available at http://www.nist.gov.

#### Dynamic light scattering

Particles were dispersed in nanopure water. Hydrodynamic size distributions in particle-suspensions were analyzed by Dynamic Light Scattering (DLS), on a Malvern Zetasizer Nano ZS (Malvern Instruments Ltd., UK). The distributions were determined directly in the instillation solutions in 1 ml polystyrene cuvettes at 25 °C. Six repeated measurements on the same sample were carried out and averaged. For the calculation of hydrodynamic size, the refractive (R_i_) and absorption indices (R_s_) of carbon black Printex90 of 2.020 and 2000 were applied for all particles, with standard optical and viscosity properties for H_2_O.

#### PAH contents

PAH contents were evaluated by GC-MS and extracted with cyclohexane from the Nanopure water suspensions of each particle [69].

#### Metal contents

*Sample preparation*: As it was not possible to transfer the amount of 4 mg airport particle matter from the collection flasks to vials for microwave-assisted acid digestion, a volume of 1 mL of 25% (v/v) nitric acid was directly added to the flasks for acid extraction. Additionally, NIST2975 and CB were included in the analysis. For the preparation of these samples, approximately 1 mg of material were weighed into 13 mL polypropylene tubes (Sarstedt, Nümbrecht, Germany) and 1 mL of 25% (v/v) nitric acid added. All samples very gently agitated for 30 min to assure the dispersion of the particles. Afterwards, the flasks and tubes were transferred to a shaker (Stuart Scientific SF1) and agitated at 600 oscillations per min for 30 min. After incubation for approximately 72 h at room temperature without agitation, the samples were placed in the shaker for another 24 h and finally transferred with 6 mL of ultrapure water into polypropylene tubes. An empty flask (same type as used to collect the airport particles) and polypropylene tubes (as used for NIST2975 and CB) were treated in the same way as the samples to obtain suitable blank solutions.

*Analysis*: Before analysis, the samples were centrifuged for 5 min at 4500 x g (Heraeus Multifuge X3 FR, Thermo Scientific), because no complete digestion of the particles was achieved. A volume of 5 mL of the supernatant was transferred to a new polypropylene tube and 0.05 mL of 100 ng/mL rhodium (Rh) solution added as internal standard. The samples were further diluted 5- or 100-fold with 5% nitric acid. A triple quadrupole inductive coupled plasma mass spectrometer (ICP-MS) (Agilent 8900 ICP-QQQ, Santa Clara, USA) equipped with a MicroMist borosilicate glass concentric nebulizer and a Scott type double-pass water-cooled spray chamber was run in no gas (Cd, Hg, Pb, Bi, U) or helium (remaining elements) mode with 0.1–3 s integration time per mass. The following plasma parameters were used: 1550 W RF power, 15 L min^− 1^ plasma gas, 0.9 L min^− 1^ auxiliary gas and 0.99 L min^− 1^ nebulizer gas. The cell gas flow in helium mode was 5 mL min^− 1^. The auto sampler (SPS4, Agilent Technologies) introduced the samples into the ICP-MS with a sample uptake time of 30 s (0.5 rps) and a stabilization time of 30 s (0.1 rps). Quantification was performed based on external calibration (multi-element standards of 5, 10, 25, 50 and 100 μg L^− 1^; for mercury 0.5, 1.0, 2.5, 5.0 and 10 μg L^− 1^) with internal standardization (1 μg L^− 1^ Rh). As quality control, a mixture of 1 μg L^− 1^ Li, Ba, Bi, V and As was analyzed.

### Mice

A total of 212 female C57BL/6Tac mice 7 weeks old at arrival (BW at instillation: 19 ± 1.1) were used in this study. The mice were group-housed in standard cages with 6–8 mice with ad libitum access to tap water and Altromin 1324 rodent diet, and provided with saw dust bedding, mouse house, wooden chew blocks and Enviro Dri nesting material. The mice were kept at 21 ± 1 °C and 50 ± 10% humidity and a 12 h light-dark circle.

#### Study design

After 1 week of acclimatization, mice were exposed to a single dose of collected particles of either 6 μg, 18 μg or 54 μg per mouse by intratracheal instillation (6–8 mice per dose per particle exposure) in three different exposure series. For each euthanization date, all vehicle control mice were pooled together into one control group: e.g. for day 90 exposures there were six different euthanization dates, hence there were in total 12 vehicle control mice. Across doses and time points, 52 mice were used for JEP, 51 mice for CAP, 50 mice for NIST2975, 18 mice for CB, and 41 vehicle control mice. On day 28 and day 90, five of these mice per treatment were used separately for histology (no histology was performed on CB instilled mice).

#### Instillation procedure

JEP, CAP, NIST2975, and CB were prepared as previously described [23]. Briefly, particles were suspended in Nanopure Diamond Water and sonicated for 16 min using a Branson Sonifier S-450D (Branson Ultrasonics Corp, Danbury, CT, USA). The suspensions were diluted and the dilutions were re-sonicated for 2 min. Nanopure Diamond Water was prepared similarly as vehicle. All solutions were freshly prepared and instilled within 1 h.

Instillation procedure was carried out essentially as described by others [70]. Intratracheal instillation procedure: A syringe was prepared with correct instillation dose in 50 μl vehicle located at the top and 200 μl air located after the instillation volume, to ensure maximum delivery into the lung. One cage of mice was simultaneously placed in an anesthesia box, and induced with 4% isoflurane and subsequently maintained at 2.5% isoflurane. In preparation for instillation, one mouse at a time was fixated by the front teeth in a customized fixation bracket on a 40-degree sloped platform with back support. A diode light was placed at the larynx visualizing the breathing pattern. With a blunt non-harmful forceps the tongue was grabbed and pressed towards the lower jaw by a small spatula in the opposite hand, to expose the pharynx. The trachea was then intubated using a 24-gauge BD Insyte catheter (Ref: 381212, Becton Dickinson, Brøndby, Denmark) with a shortened needle. Upon placement of the catheter, the spatula holding the pharynx was removed. To ensure correct location of the catheter, a small but highly sensitive pressure transducer was placed at the top of the catheter (developed by our laboratory in collaboration with John Frederiksen (FFE/P, Copenhagen, Denmark). When the catheter was correctly placed, this was indicated by a clicking sound triggered by the pressure variation as air was inhaled and exhaled, and the mouse was instilled. The catheter and syringe was removed, and the mouse was carefully shaken twice, fully cupped and secured in one hand, to ensure confinement of the instilled material in the lungs and spreading downwards towards the alveoli. The mouse was then returned to its home cage, placed on a heating plate, to ensure optimal recovery from anesthesia. The entire procedure took < 1 min per mouse. The mice were weighed afterwards. The mice were all observed and evaluated for signs of discomfort immediately after anesthetic seponation, and evaluated frequently until euthanization, by visual inspections and body weight monitoring. Humane endpoints were weight loss of maximum 20%, clear signs of discomfort such as ruffled fur, isolation, facial pain expression, and changed respiration.

#### Organ harvest and preparation

For bronchoalveolar lavage (BAL), the mice were anesthetized with 25 mg/ml tiletamin and 25 mg/ml zolazepam (Zoletil™ Vet. 250 mg, Virbac), xylaxin (Rompun™ Vet. 20 mg/ml, Bayer), and fentanyl 50 mg/ml in sterile saline. The lungs were flushed twice with 1 ml sterile saline per flush to obtain BAL fluid. BAL fluid was kept on ice and centrifuged at 400 G at 4 °C for 10 min within 1 h. The supernatant was allocated into smaller lots, snap-frozen and stored at − 80 °C for further processing. The BAL cell pellet was further processed for automated total cell count (NucleoCounter NC-200TM, Chemometec, Denmark) following manufacturer’s protocol, and manual differential count of inflammatory cell subsets, or further processed for Comet assay. The still sedated mice were euthanized by heart-puncture and blood was collected in EDTA tubes and plasma was stored at − 80 °C. Lung and liver tissue were harvested for extraction of RNA, mRNA expression, genotoxicity determination by comet assay, and histopathology for which kidney and spleen were harvested as well. BAL samples and samples for Comet assay were prepared and analyzed as previously described [23, 46]. Saa mRNA (Taq-Man Reverse Transcriptation Reagent Kit and RTqPCR on ViiA™7, ThermoFischer Scientific, Denmark) and SAA3 plasma protein (Mouse SAA-3 ELISA, EZMSAA3-12 K, Merck Millipore, Denmark; Epoch™ microplate spectrophotometer, BioTek, Winooski, USA) were prepared and measured according to manufacturer’s protocols and lung and liver tissue was prepared and dyed for histopathological examination, as previously described [46].

#### In vivo data statistics

Statistical analysis was performed in GraphPad Prism (GraphPad Prism, version 7.03 for Windows, GraphPad Software, La Jolla California USA, www.graphpad.com). Data was assessed for normality, variation and outliers by inspection of scatter plots and by statistical evaluation (Brown-Forsythe F-test for variance and ROUT for outliers, provided by GraphPad Prism). Serum Amyloid A data was log2 transformed to achieve equal variance and normalization. Due to abundance in values equal to zero, log transformations were not applicable for BAL data. Due to sample sizes, outliers were included and depicted on figures; however, data was analyzed with and without outliers and reported in-text if deviant. Data following the Gaussian distribution and equal variance assumptions was analyzed by one-way ANOVA followed by Dunnett’s (comparison to control group) or Sidak’s multiple comparison test (pre-selected column pairs). Nonparametric data was analyzed by Kruskal-Wallis followed by Dunn’s multiple comparisons test. The following comparisons were made: 1) Exposure groups compared to vehicle control group (reported as asterisks on figures) 2) Exposure groups compared to CB benchmark particle exposure group (reported in data tables) 3) Exposure groups compared to standard diesel particle exposure groups (NIST2975 and published data on NIST1650) (reported in data tables). Increasing dose-response effects were confirmed with test for linear trend, where the *alerting R*^*2*^ (referred to as R^2^ in the text) is the fraction of the variance between group means that is accounted for by the linear trend (Altman/Sheskin, provided by GraphPad Prism).

## Additional files

Additional file 1: **Figure S1 a.** Illustrations of measurement strategies (figure). **b.** Jet engine test facility measurement (figure and text). **c.** Aerosols characterized by EM of impactor samples (text and images). **d.** Dynamic Light Scattering (text and figure). **e.** EDS analysis (images). (PDF 2847 kb)

Additional file 2: **Figure S2 a.** Scatter plots of BAL fluid cells on day 1, 28, and 90 post-instillation, eosinophil influx, and BET area vs neutrophil influx (figures). **b.** Saa3 in lung and liver on day 28 and 90 (figure). **c**. % DNA in comet tail and DNA strand breaks (figure and table). (PDF 1431 kb)

## Data Availability

The datasets used and/or analyzed during the current study are available from the corresponding author on reasonable request.

## Abbreviations

BAL: Broncho-Alveolar Lavage
CAP: Commercial Airport Particles
CB: Carbon Black
DLS: Dynamic Light Scattering
ECD: Equivalent Circular Diameter
ELPI: Electrical Low Pressure Impactor
ESP: Electrostatic Precipitator
FT: Fuel Truck
GC-MS: Gas Chromatography Mass Spectrometry
ICP-MS: Inductive Coupled Plasma Mass Spectrometry
JEP: Jet Engine Particles
NIST: National Institute of Standards and Technology
PA: Plane Arriving
PAH: Polycyclic Aromatic Hydrocarbons
PL: Plane Leaving
SAA: Serum Amyloid A
SEM: Scanning Electron Microscopy
SRM: Standard Reference Material
TEM: Transmission Electron Microscopy
UFP: Ultrafine Particles
