# Supplementary material for: Airport emission particles: exposure characterization and toxicity following intratracheal instillation in mice
Source: Part Fibre Toxicol. 2019 Jun 11;16:23. doi: 10.1186/s12989-019-0305-5 (PMC6558896; doi:10.1186/s12989-019-0305-5)
Supplement: Supplementary file 1 — Figure S1 a. Illustrations of measurement strategies (figure). b. Jet engine test facility measurement (figure and text). c. Aerosols characterized by EM of impactor samples (text and images). d. Dynamic Light Scattering (text and figure). e. EDS analysis (images). (PDF 2847 kb) [file 12989_2019_305_MOESM1_ESM.pdf]

Additional File S1 A

Illustrations of measurement strategies

Figure

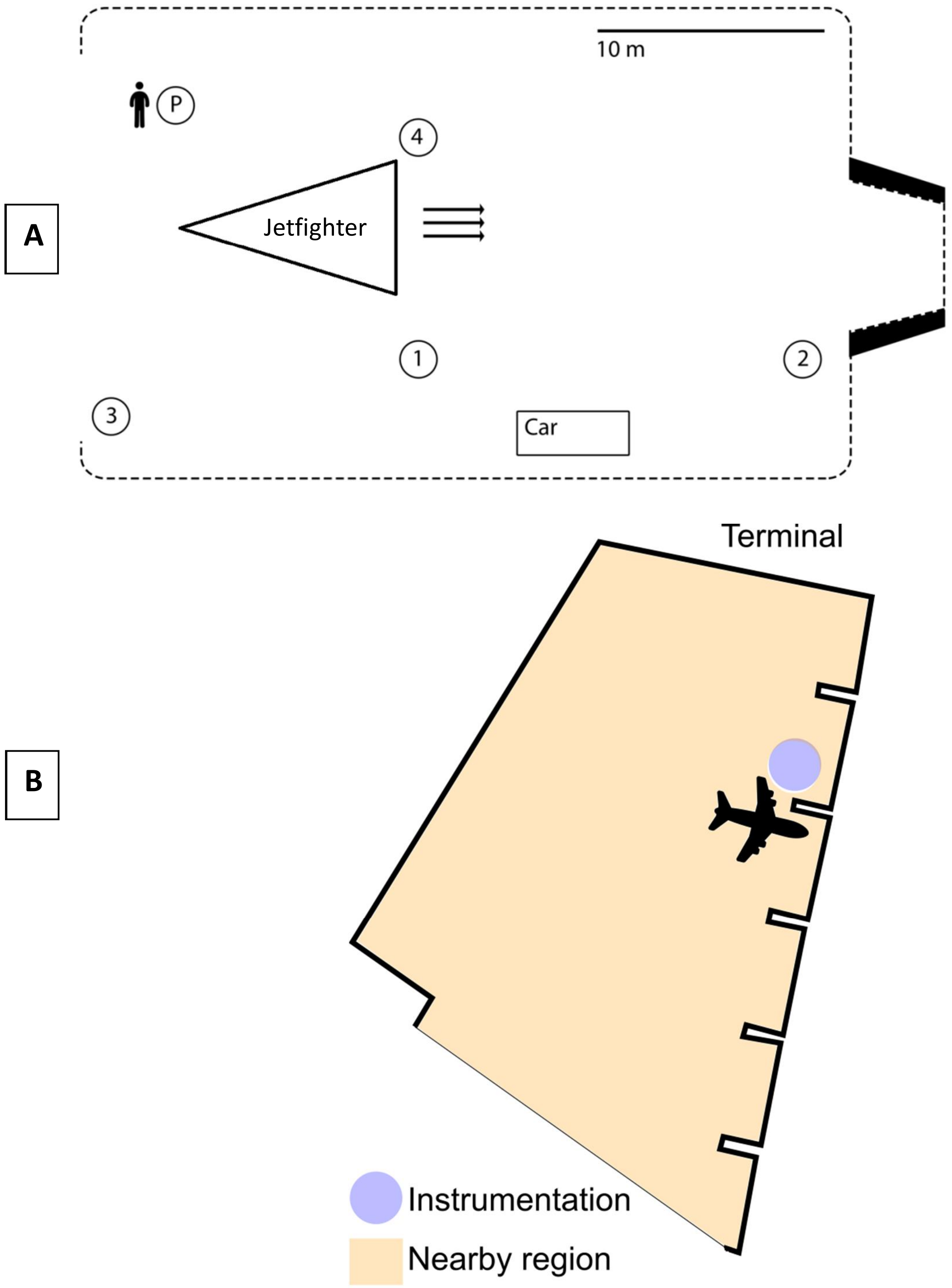

Fig. S1A. A: Schematic illustration of the measurement strategy in a jetfighter shelter at a non-commercial airfield, during collection of particles. Sampling stations were placed to measure the airborne particle concentrations in the near field, far field and in the breathing zone of the flight personnel. The illustration shows exact size ratios of the measuring zone and location of instruments. Position 1: Real-time particle monitoring with an Electrical Low Pressure Impactor (ELPI), Positions 2-4 and P (personal breathing zone): particle number concentration, mean particle size and lung-deposited surface area (LDSA) measured with four DiSCminis. Additionally, at position 3 there was a Nanoscan for size-dependent particle number measurements and an Optical Particle Counter (OPC). The plane is positioned in the arrived state and will exit through the open front (left) upon preparing for takeoff. The hangar roof forms a half elliptical volume of 4721 m^3. B: Placement of instruments at the apron of a commercial airport.

Additional File S1 B

Jet engine test facility measurement

Figure and text

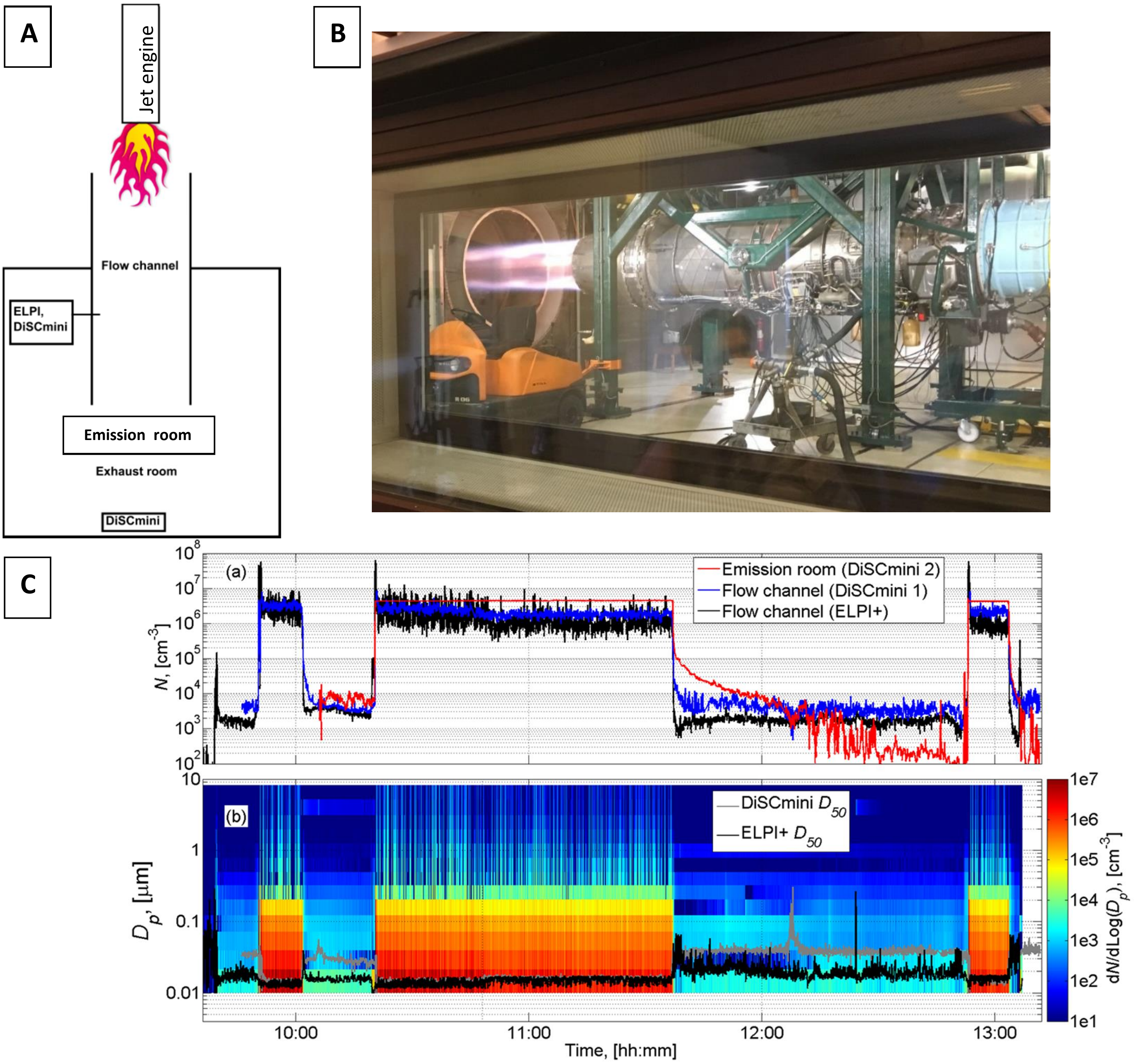

Fig. S1B. A: Layout of the jet engine test facility. B: Testing the engine. C: Particle number concentrations measured by the ELPI+ and DiSCmini's during three test runs.

Jet engine test facility

Particle number concentrations were measured during three engine tests at unknown power level from a flow channel using DiSCmini and ELPI+ (*ca.* 5 cm from the wall) and exhaust room (Fig. S1B). Concentration at the flow channel was ca. 2×10^6 1/cm^3, which was smaller than concentration measured from the exhaust room (>4×10^6 1/cm^3) (Fig. S1B, C (a)). This was probably due to incomplete mixing of concentrations in the flow channel. During the engine running, the average geometric mean diameter (D_{50}) of the ELPI+ and DiSCmini were 15 nm and 15.4 nm, respectively, and the average *LDSA* concentration was 1600 µm^2/cm^3 in the flow channel and >7900 µm^2/cm^3 in the emission room (Fig. S1B, C (b)).

Additional File S1 C

Aerosols characterized by EM of impactor samples

Text and Images

Description of impacted aerosols by electron microscopic analysis

JEP: Two different JEP impactor samples and a single CAP impactor sample from the apron of the commercial airport were acquired.

The low number density observed on the grids even after 60 seconds of sampling shows that the background aerosol contains only very few particles (results not shown), and can be completely neglected when analyzing the take-off sample, which was collected for 5 seconds. The TEM grids from the first and second stage of the take-off sample were densely populated with highly agglomerated soot particles ranging from approximately 500 nm to tens of micrometers in equivalent circular diameter (ECD) (Fig. S1C, A). Due to the high loadings on the grids, it was not possible to determine whether the large soot agglomerates were a result of co-deposition during sampling, or whether they were airborne as agglomerates. The primary particle sizes of the agglomerated particles ranged from approximately 10 to 30 nm. The elemental composition of selected particles was investigated via EDS analysis, which showed only carbon and oxygen peaks in ratios of 10:1 or higher. The lowest stage of the impactor was also densely populated by soot agglomerates, with the largest measuring a few micrometer in ECD. However, the grid also contained areas with much smaller agglomerates, which were analyzed at higher magnification using the TEM. No single primary particles were found on the grid but agglomerates down to 30 nm in ECD were observed, which contained as few as three primary particles. However, these areas of the sample were dominated by 50-200 nm agglomerates with 10 to 100 primary particles (Fig. S1C, B). When investigating the primary particles at higher magnification the typical soot structure with fringes of graphene-like flakes was clearly visible on top of the amorphous Formvar structure of the substrate (Fig. S1C, C).

CAP: A single CAP impactor sample was collected for 30 seconds at the sampling site on the apron of the commercial airport.

The first stage contained many micrometer-sized particles ranging between 1 and 50 µm. The particles were mainly rectangular or square, consistent with the shape of salt crystals, which was confirmed by EDS-analysis where sodium, carbon, oxygen, calcium, chloride and sulfur were the main elements. A few micrometer-sized particles, which appeared to be pollen, were also observed on the grid, but salts were the dominating particle type. This was expected, as the commercial airport is situated in close proximity of the sea. The second stage contained only very few particles, which were in the size range between 500 nm and 1 µm in ECD. The last stage of the impactor displayed an area covering approximately 12 grid squares, which was densely populated with particles. The size of the

impact area was consistent with the size of the impactor orifice. Within the area, particle sizes varied from approximately 1 µm to a few nm in ECD. Particles were often found in a circular deposition pattern where many small particles surround a larger one, with the size of the surrounding particles decreasing with distance from the center particle (Fig. S1C, D and E). These circular patterns often occur when droplets are impacted. Once the liquid in the droplets evaporate, which could be either during impaction itself or in the vacuum of the microscope, particles contained in the droplet are left behind in the observed circular pattern. The dominating particle type was salt particles, with carbon, oxygen, sodium, sulfur, potassium, calcium, magnesium, and chloride as the main elements. Soot particles were also observed on the grid, which was confirmed by TEM, where the clear onion-like structure of soot particles was visible on top of the amorphous substrate (Fig. S1C, F). As seen in figure E and F, soot particles were found in (three) different states: as free, individual agglomerates, as well as agglomerated to other particles (e.g. larger particles, salts, and others) and, as the above described findings suggests, associated with or captured in droplets. The surrounding spherical particles, marked with red arrows were interpreted to be small salt condensates. These evaporated quickly under the electron beam and structural analysis proved they were not soot particles. This observation was general for all the small spherical particles deposited near the edge of the circular deposition patterns (Fig. S1C, D-F). These particles could therefore be formed from low volatile compounds and salt dissolved in the initial droplet, which have sufficiently low vapor pressures to avoid evaporation under the vacuum of the microscope, leaving behind small liquid particles as the main content of the droplet evaporates.

<u>Images below: EM images of impactor-collected material</u>

Fig. S1C. Electron micrographs of particles collected at a non-commercial airfield shelter (JEP) and from the apron of a commercial airport (CAP) from direct impactor-collection. Two different JEP impactor samples were acquired, each with a TEM grid installed on all three stages. One sample was collected when no planes were active to give an indication of the background aerosol. The other sample was obtained through the window of a car driving behind a jetfighter during taxi out. The background aerosol was sampled for 60 seconds, while the other sample was collected for 5 seconds, due to the high number concentrations detected by the online instrumentation (Article, Table 1).

A single CAP impactor sample was collected for 30 seconds at the sampling site on the apron of the commercial airport in the proximity of an aircraft during taxi, with TEM grids installed on all three stages. The grids were all analyzed with SEM, while only the lowest stage was examined by TEM.

A: Dense population with highly agglomerated soot particles ranging from approximately 500 nm to tens of micrometers. B: Higher magnification showing smaller soot agglomerates. C: Details of the typical soot structure with fringes of graphene-like flakes visible on top of the amorphous Formvar structure D: Particles were often found in a circular deposition pattern, with the size of the particles decreasing with distance from the center particle. E: Higher magnification showing detail of droplets. F. Details of the clear onion-like structure of soot particles visible on top of the amorphous substrate.

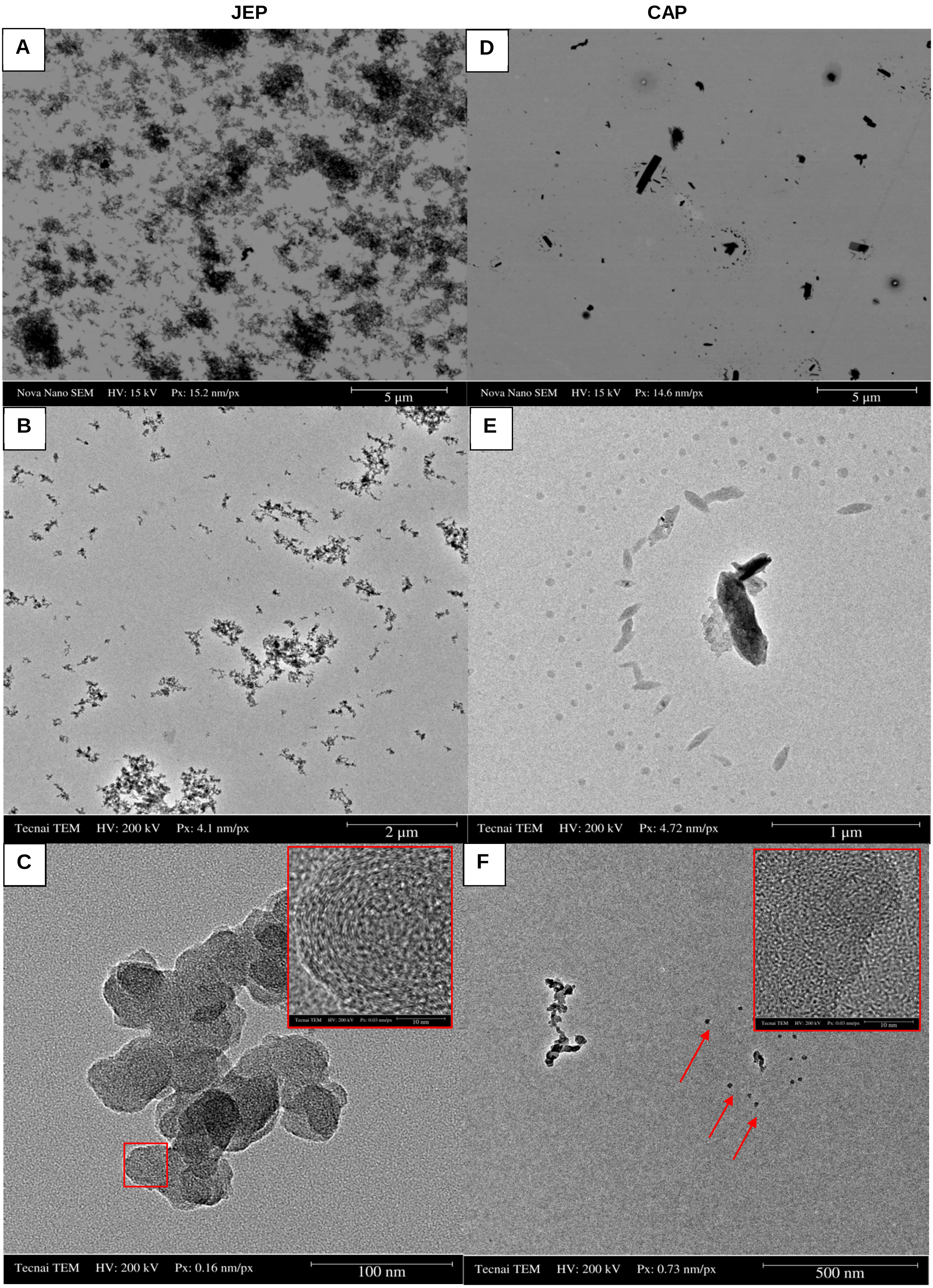

S1C. Electron micrographs of airport-collected particles. See description above.

Additional File S1 D
Dynamic Light Scattering
Text and Figure

<u>Description of Dynamic Light Scattering</u>
All particles were dispersed in Nanopure water and sonicated to obtain stable dispersions (Hadrup et al. Mutagenesis 2017). The hydrodynamic number size distribution and intensity were measured by Dynamic Light Scatter (DLS) for particle concentrations 3.24 mg/ml, 1.08 mg/ml, 0.36 mg/ml and 0.12 mg/ml, corresponding to 162, 54, 18 and 6 µg particulate matter per mouse (Fig. S1E). For JEP and CAP, 54, 18 and 6 µg were used, whereas 162, 54 and 18 µg were used for NIST2975 to match the previously published dose range for NIST1650b (Kyjovska et al. Mutagenesis 2015). CB was included at a single dose level of 54 µg. For JEP dispersions, the number distribution showed bimodal peaks in the size range of 10-100 nm (Fig. S1E A). Similar patterns were observed for CAP (Fig. S1E B). CAP and JEP both had a small fraction of larger particles outside the range of measurement (Fig. S1E A+B, Intensity). CB peaked at 50.75 nm (Fig. S1E C) and NIST2975 peaked at 50-60 nm (Fig. S1E D). The Z-average particle size varied from 136-269 nm for CAP and from 143-196 nm for JEP, depending on concentration (main article, Table 4).

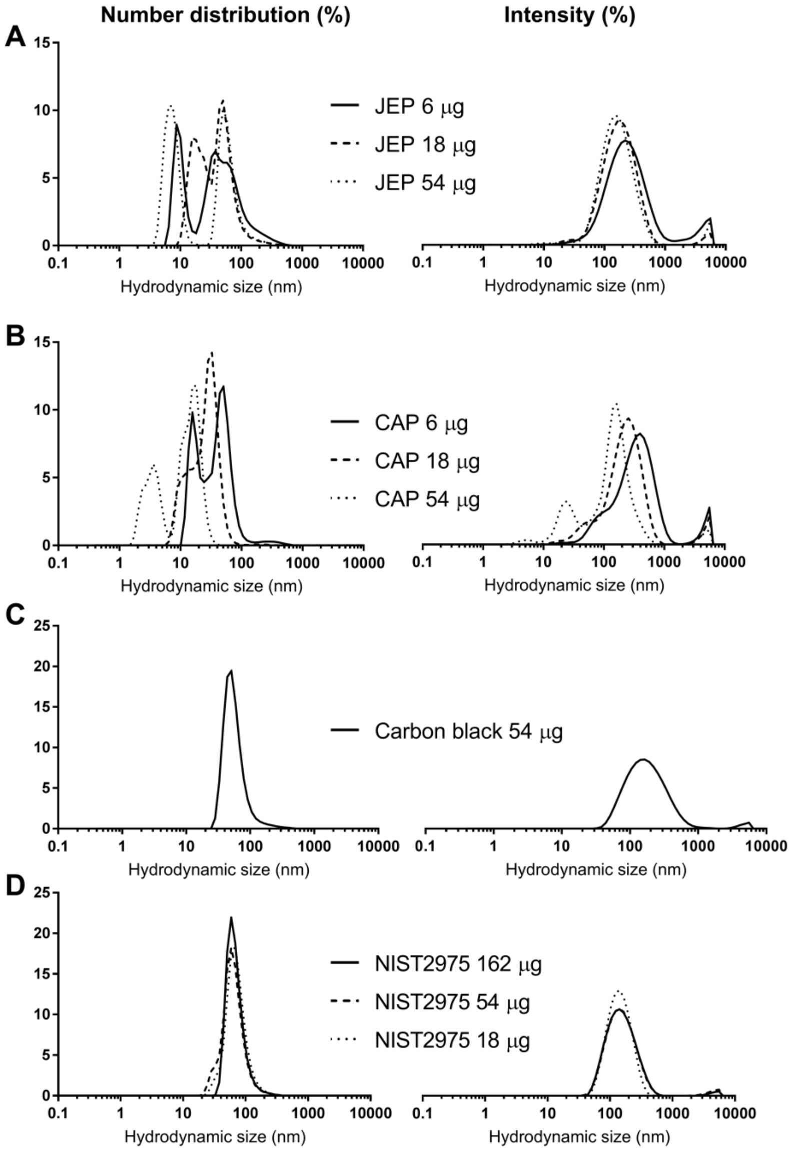

Fig. S1 D. Dynamic Light Scattering analysis of hydrodynamic particle size, shown as intensity and number distribution of particles dispersed in water. A: jet engine particles (JEP). B: commercial airport particles (CAP). C: Carbon black Printex90 (CB). D: NIST2975.

Additional File S1 E
EDS analysis
Images

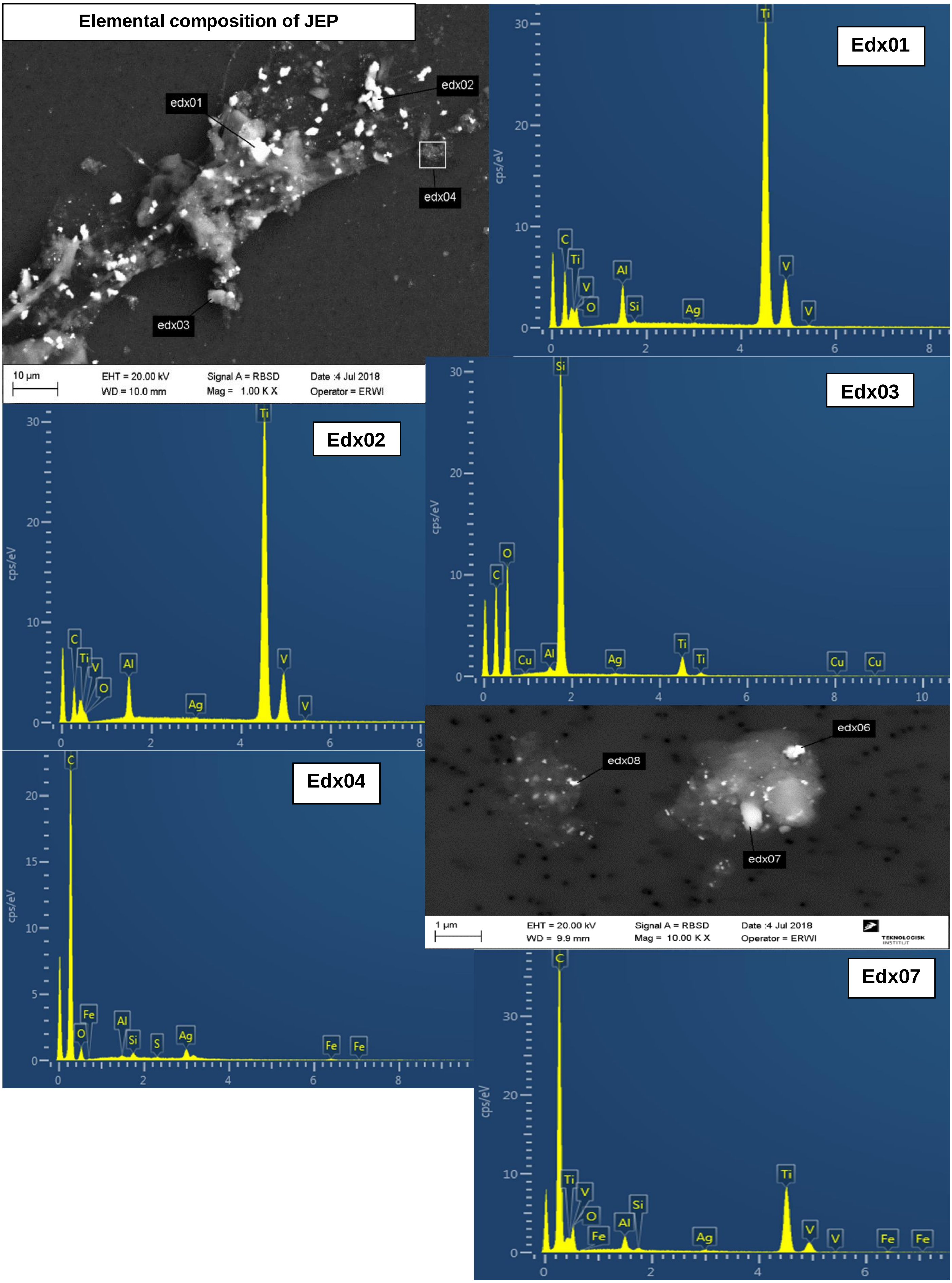

S1E (1). Electron micrographs with EDS analysis of particles collected at a non-commercial airfield shelter (JEP) (particles in suspension).

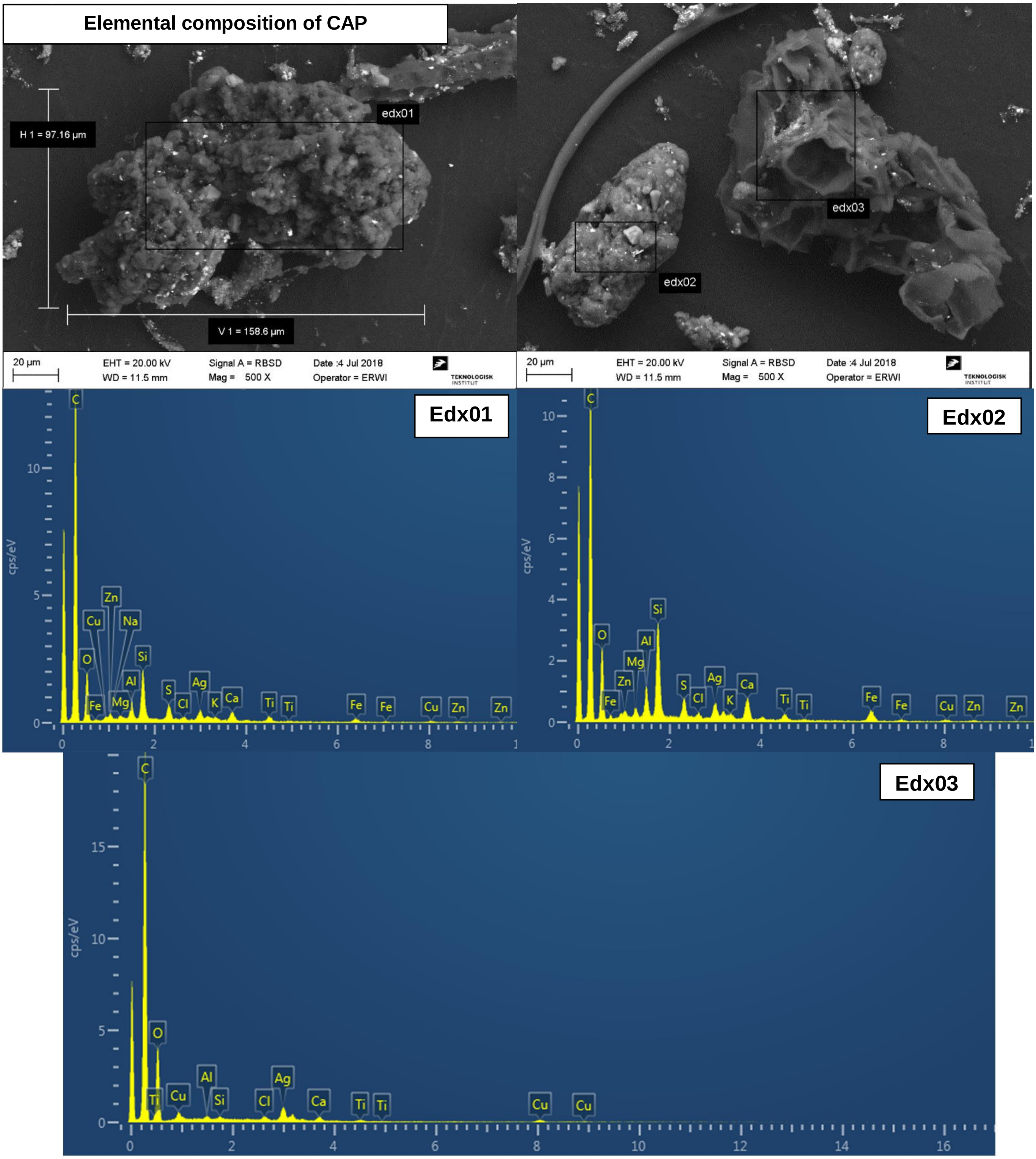

S1E (2). Electron micrographs with EDS analysis of particles collected at the apron of a commercial airport (CAP) (particles in suspension).

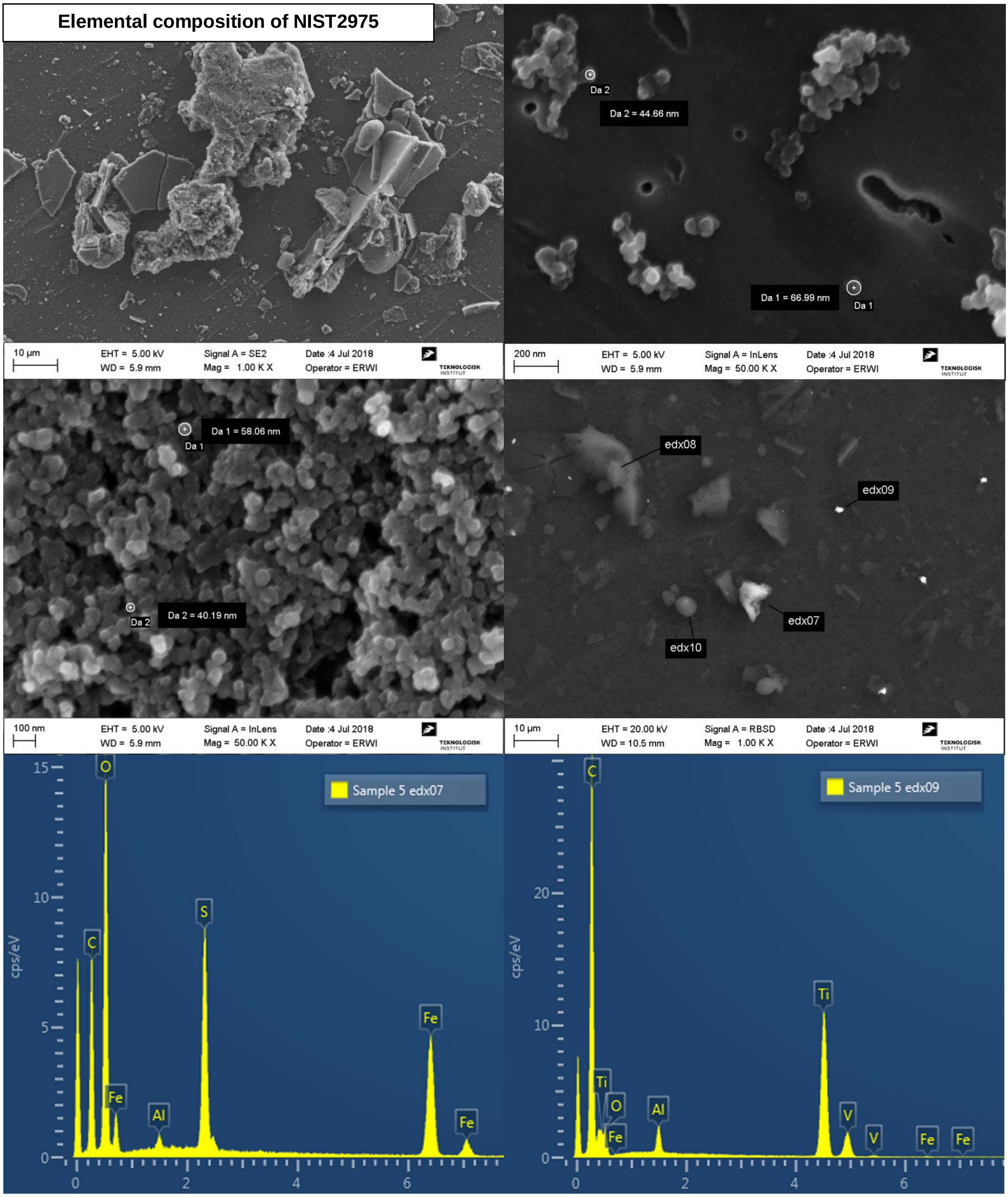

S1E (3). Electron micrographs with EDS analysis of standard reference particles NIST2975 (particles in suspension).
