# Supplementary material for: Airport emission particles: exposure characterization and toxicity following intratracheal instillation in mice
Source: Part Fibre Toxicol. 2019 Jun 11;16:23. doi: 10.1186/s12989-019-0305-5 (PMC6558896; doi:10.1186/s12989-019-0305-5)
Supplement: Supplementary file 2 — Figure S2 a. Scatter plots of BAL fluid cells on day 1, 28, and 90 post-instillation, eosinophil influx, and BET area vs neutrophil influx (figures). b. Saa3 in lung and liver on day 28 and 90 (figure). c. % DNA in comet tail and DNA strand breaks (figure and table). (PDF 1431 kb) [file 12989_2019_305_MOESM2_ESM.pdf]

Additional File S2 A

Scatter plots of BAL fluid cells on day 1, 28, and 90 post-instillation

Figures

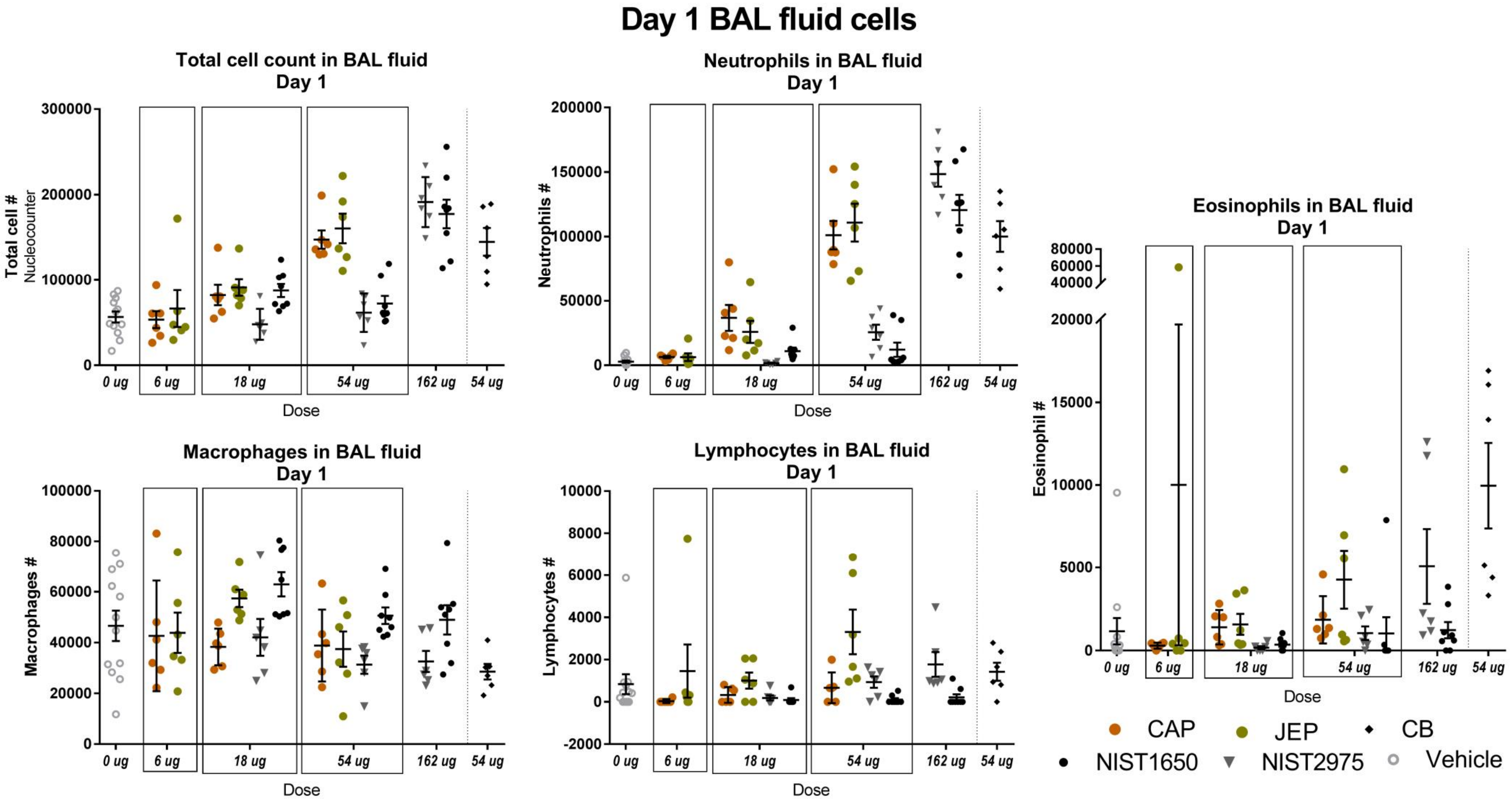

Fig. S2A (1). Cells in BAL fluid on day 1 following exposure to jet engine particles (JEP), commercial airport particles (CAP), and reference particles NIST2975, NIST1650, and Carbon black Printex90 (CB) (scatter plots, mean+SEM). Mice were exposed to 6, 18, and 54 µg of JEP and CAP, to 54 µg of CB, and to 18, 54, and 162 µg of NIST particles with 6 mice in each group. Data for NIST1650 was obtained from a previously published study (Kyjovska et al. Mutagenesis 2015).

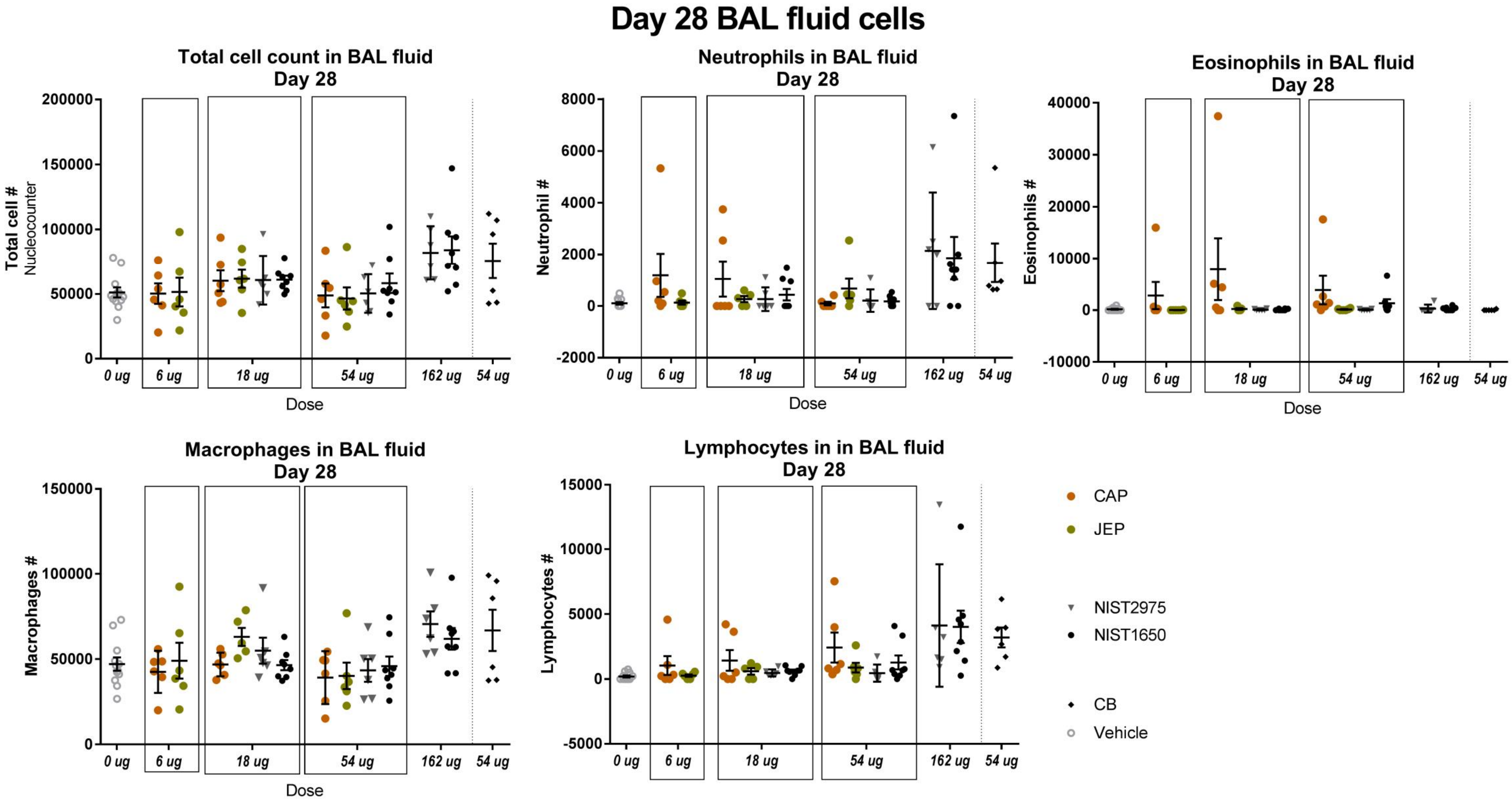

Fig. S2A (2). Cells in BAL fluid on day 28 following exposure to jet engine particles (JEP), commercial airport particles (CAP), and reference particles NIST2975, NIST1650, and Carbon black Printex90 (CB) (scatter plots, mean+SEM). Mice were exposed to 6, 18, and 54 µg of JEP and CAP, to 54 µg of CB, and to 18, 54, and 162 µg of NIST particles with 6 mice in each group. Data for NIST1650 was obtained from a previously published study (Kyjovska et al. Mutagenesis 2015).

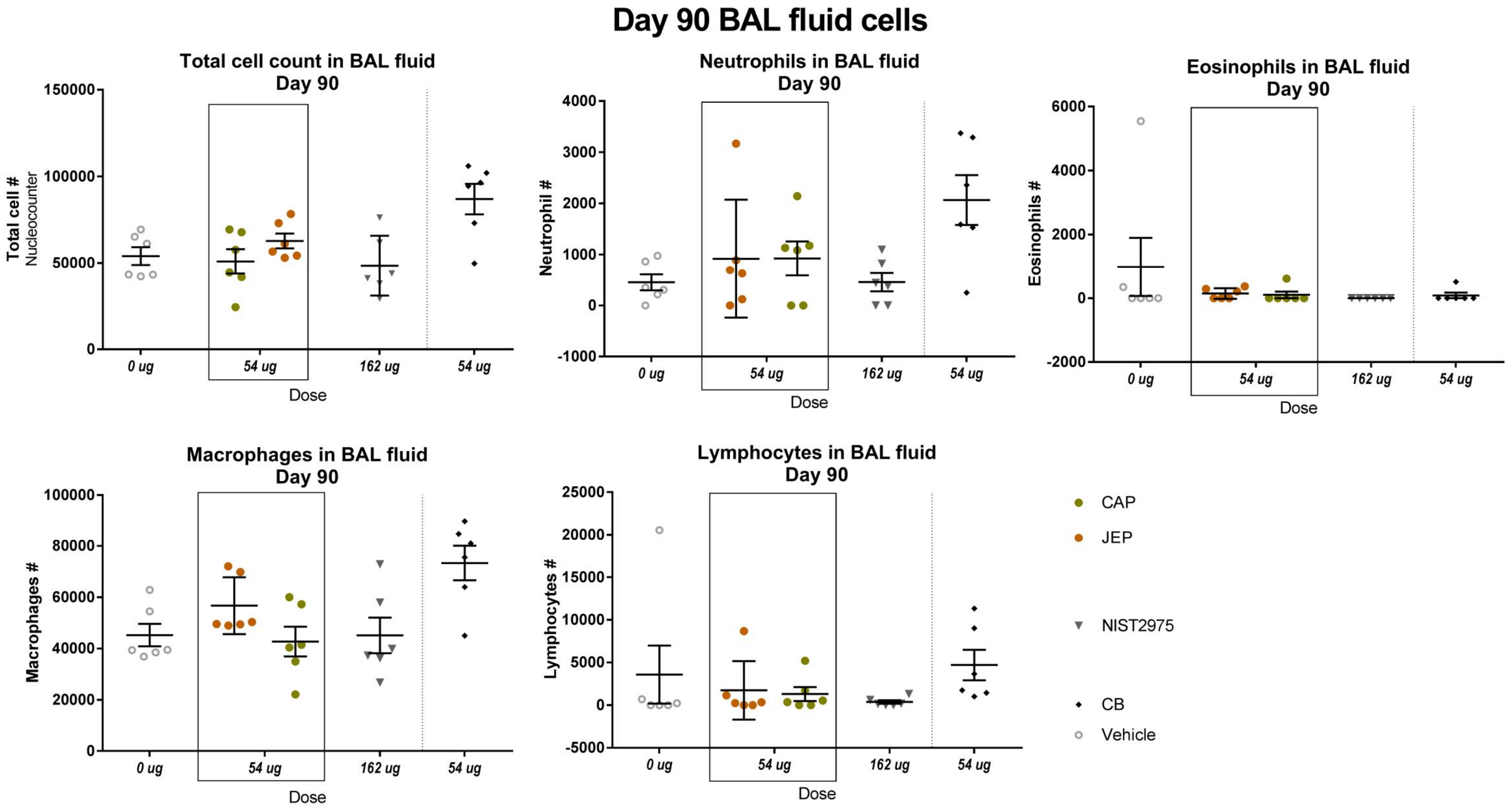

Fig. S2A (3). Cells in BAL fluid on day 90 following exposure to jet engine particles (JEP), commercial airport particles (CAP), and reference particles NIST2975, NIST1650, and Carbon black Printex90 (CB) (scatter plots, mean+SEM). Mice were exposed to 6, 18, and 54 µg of JEP and CAP, to 54 µg of CB, and to 18, 54, and 162 µg of NIST particles with 6 mice in each group. Data for NIST1650 was obtained from a previously published study (Kyjovska et al. Mutagenesis 2015).

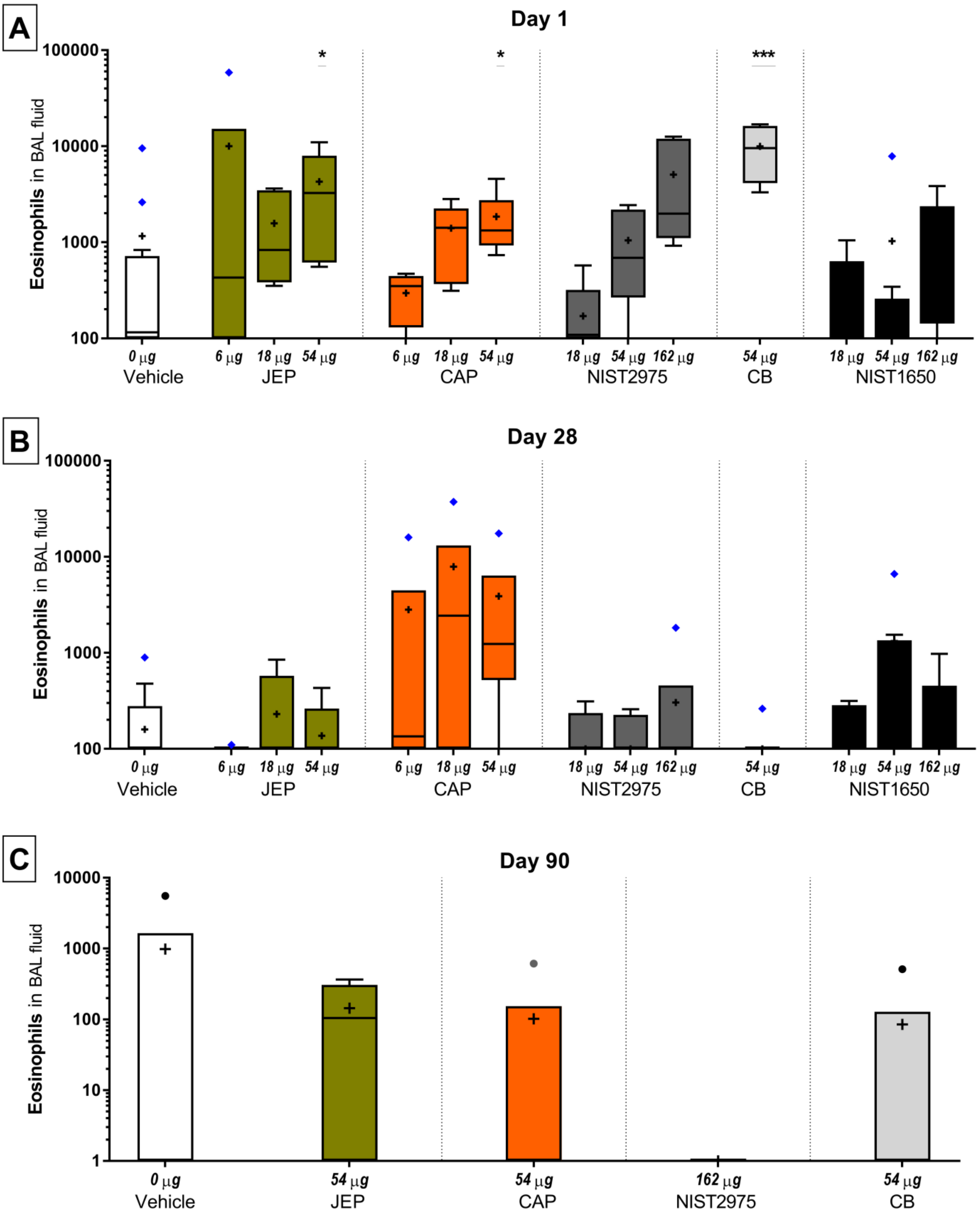

Fig. S2A (4). Eosinophil influx in BAL fluid on day 1, 28, and 90 following exposure to jet engine particles (JEP), commercial airport particles (CAP), and reference particles NIST2975, NIST1650, and Carbon black Printex90 (CB) (Tukey plots, +: mean, line: median, diamonds: outliers). Mice were exposed to 6, 18, and 54 µg of JEP and CAP, to 54 µg of CB, and to 18, 54, and 162 µg of NIST particles. Data for NIST1650 was obtained from a previously published study [19].

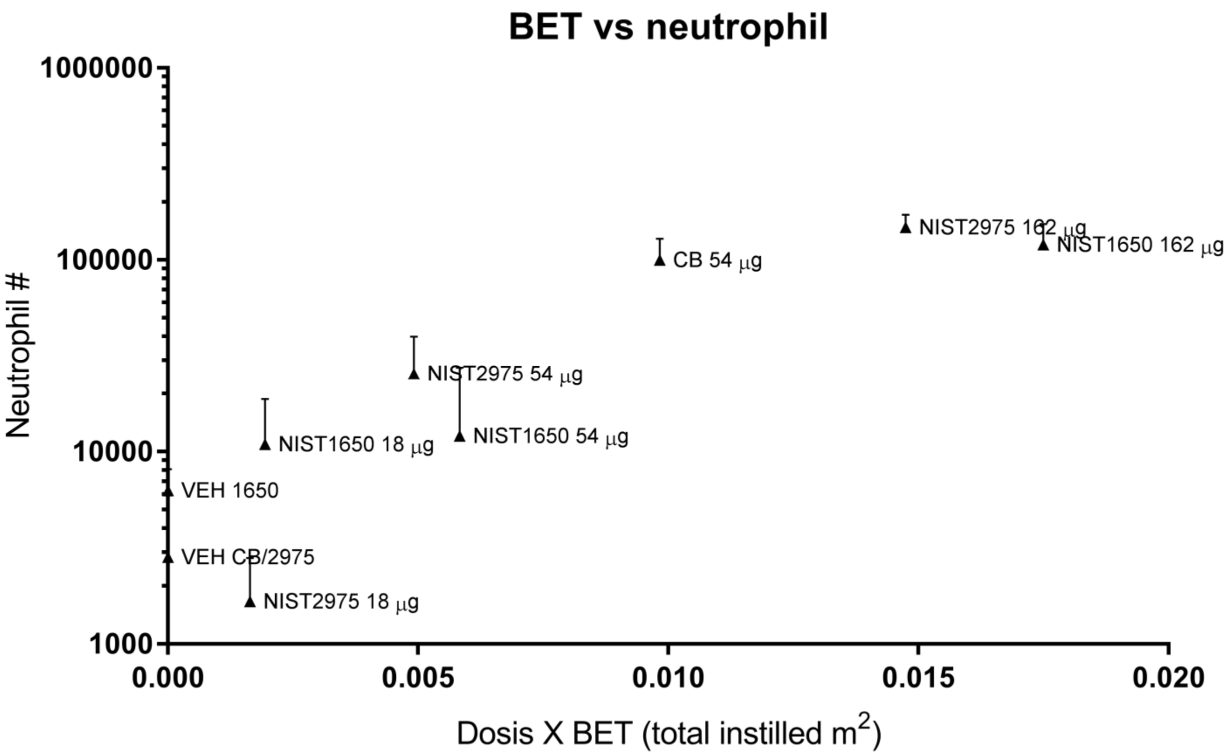

Fig. S2A (4). Surface area (BET) as predictor of NIST and CB-induced inflammation. Neutrophil influx day 1 for CB, NIST2975 and NIST1650: Mean and SD. BET x dose calculated with BET surface areas of: 182 m^2/g for CB, 108 m^2/g for NIST1650, and 91 m^2/g for NIST2975.

Additional File S2 B

Saa3 in lung and liver on day 28 and 90

Figure

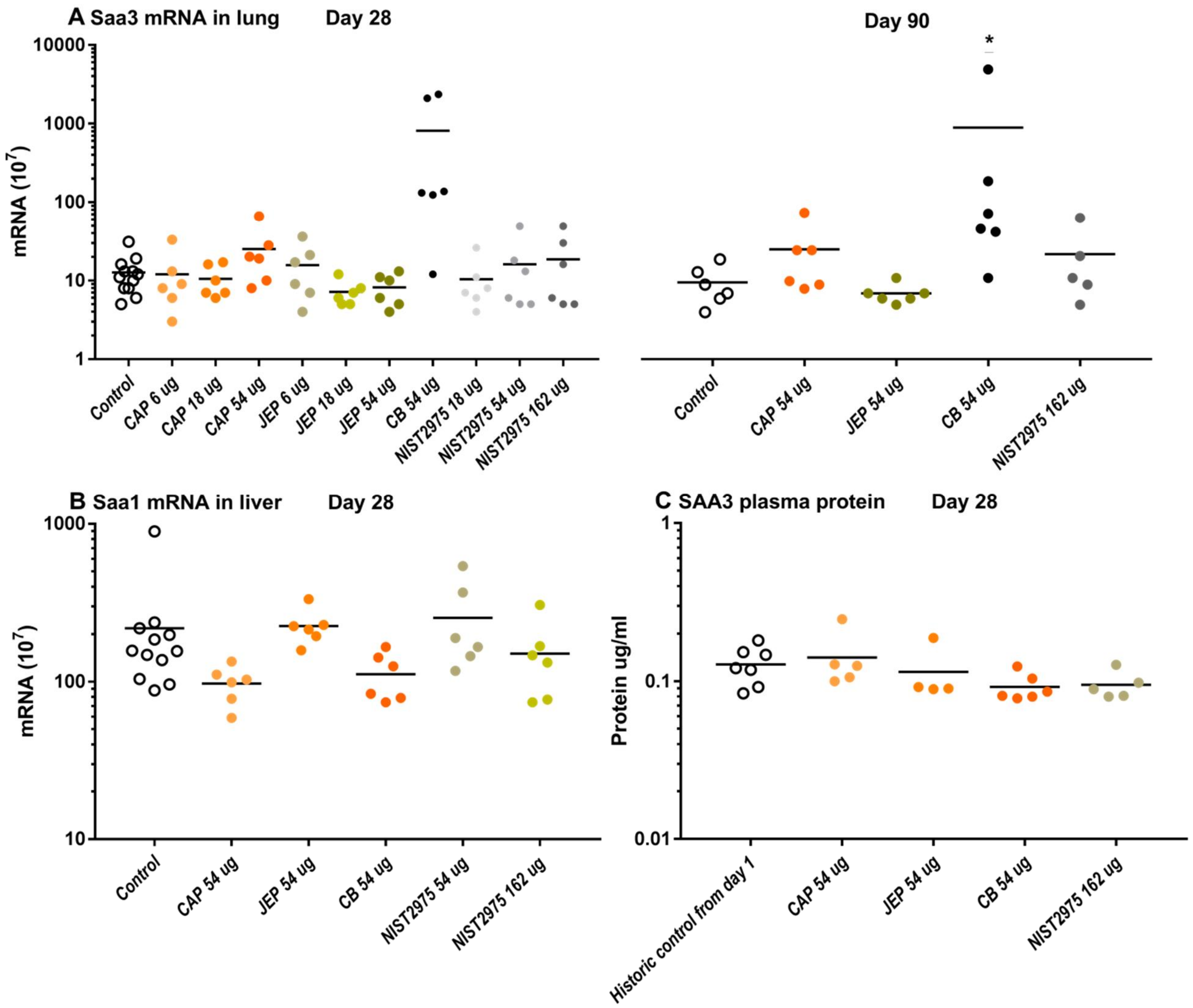

Fig. S2B. mRNA levels of Saa3 in lung on day 28 and 90, and Saa1 liver and SAA3 plasma protein on day 28 (scatter plots, mean + SEM). Saa3 mRNA in lung tissue and Saa1 mRNA in liver tissue were used as biomarkers of pulmonary and hepatic acute phase response, following intratracheal exposure of mice to particles collected at the apron of a commercial airport and in a jet shelter at a non-commercial airfield. SAA3 protein was measured in plasma. *Saa* in lung and liver was measured on day 1, 28 and 90 post-exposure, and SAA3 on day 1 and on day 28 for highest particle doses.

Additional File S2 C
% DNA in comet tail and DNA strand breaks
Figure and Table

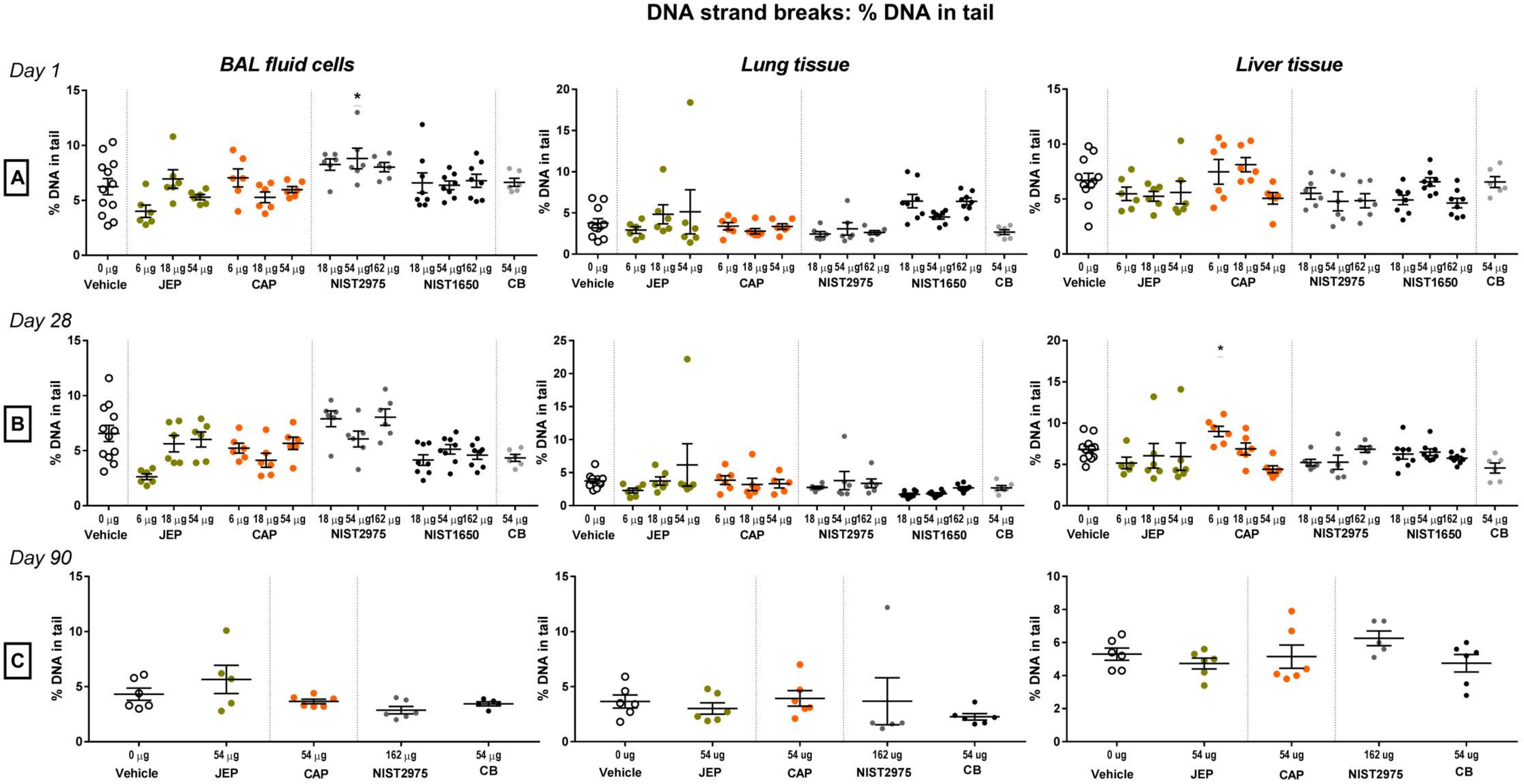

Fig. S2C. DNA strands break levels evaluated by % DNA in tail in the Comet assay on day 1, 28, and 90 following exposure to jet engine particles (JEP), commercial airport particles (CAP), and reference particles NIST2975, NIST1650, and Carbon black Printex90 (CB) (scatter plots, mean+SEM). Mice were exposed to 6, 18, and 54 µg of JEP and CAP, to 54 µg of CB, and to 18, 54, and 162 µg of NIST particles. Data for NIST1650 was obtained from a previously published study (Kyjovska et al. Mutagenesis 2015).

Table S2C. DNA damage evaluation by Comet Assay using %DNA in comet tail *(% DNA in tail)* and comet tail length (*Tail length*) in BAL, lung and liver cells from mice on day 1, 28 and 90 post-exposure to jet engine particles (JEP) collected at a non-commercial airport and to particles collected at the apron of a commercial airport (CAP).

**Day 1**	**BAL cells**		**Lung tissue**		**Liver tissue**	
	% DNA in tail	***Tail length***	***% DNA in tail***	***Tail length***	***% DNA in tail***	***Tail length***
Vehicle control	6.26±0.74	13.97±0.86	3.76±0.56	14.02±0.56	6.71±0.64	16.23±0.88
Carbon black 54 µg	6.65±0.37	15.01±0.81	2.67±0.30	12.79±0.83	6.55±0.50	15.85±0.68
CAP 6 µg	7.05±0.82	16.02±0.70	3.38±0.45	14.33±1.12	7.48±1.13	17.30±1.26
CAP 18 µg	5.28±0.49	14.46±0.58	2.78±0.33	12.04±0.71	8.13±0.65	18.04±0.72
CAP 54 µg	5.98±0.28	14.21±0.73	3.35±0.34	15.07±0.82	5.07±0.52	13.19±0.81
JEP 6 µg	4.02±0.56	11.03±0.87	2.92±0.40	12.44±0.43	5.48±0.62	14.76±0.68
JEP 18 µg	6.95±0.84	17.69±0.79(*****)	4.83±1.15	12.92±0.69	5.25±0.45	15.32±0.70
JEP 54 µg	5.30±0.24	15.34±0.63	5.13±2.68	13.35±2.06	5.60±1.03	14.66±0.86
NIST2975 18 µg	8.27±0.52	17.25±0.53(******)	2.43±0.31	12.45±0.27	5.52±0.52	14.75±0.75
NIST2975 54 µg	8.82±0.94(*****)	16.48±1.02	3.07±0.75	14.59±2.21	4.80±0.86	13.15±1.34
NIST2975 162 µg	8.03±0.42	16.57±0.69	2.62±0.25	12.67±0.68	4.85±0.64	13.20±0.89
NIST1650[a] 18 µg	-	15.74±1.23	6.44±0.82	16.51±1.27	4.93±0.44	16.84±0.81
NIST1650[a] 54 µg	-	14.97±0.44	4.50±0.26	12.42±0.59	6.56±0.38	18.16±0.78
NIST1650[a] 162 µg	-	15.95±0.99	6.40±0.42	13.77±0.92	4.64±0.43	16.15±0.82
**Day 28**	**BAL cells**		**Lung tissue**		**Liver tissue**	
	% DNA in tail	***Tail length***	***% DNA in tail***	***Tail length***	***% DNA in tail***	***Tail length***
Vehicle control	6.57±0.73	15.44±1.13	3.74±0.32	13.95±0.56	6.83±0.39	16.30±0.47
Carbon black 54 µg	4.35±0.31	12.14±0.48	2.70±0.35	10.99±0.68	4.57±0.60	12.19±0.81
CAP 6 µg	5.23±0.45	13.98±0.75	3.87±0.66	14.49±1.16	9.00±0.63(*****) **(xxx)**	19.51±0.77(*****) **(xxxx)**
CAP 18 µg	4.13±0.64	12.92±0.49	3.22±0.94	12.51±1.58	6.88±0.73**(x)**	16.52±0.45**(x)**
CAP 54 µg	5.67±0.56	14.24±0.89	3.32±0.65	13.11±1.10	4.42±0.43	12.43±0.75
JEP 6 µg	2.63±0.27	9.81±0.54	2.30±0.36	11.81±0.87	5.18±0.71	13.89±0.74
JEP 18 µg	5.63±0.74	15.78±0.60**(xx)**	3.73±0.63	12.42±1.05	6.05±1.49	16.32±1.12
JEP 54 µg	6.02±0.69	15.54±0.85**(xx)**	6.18±3.21	15.02±3.45	5.95±1.65	14.64±1.44**(x)**
NIST2975 18 µg	7.90±0.72	16.24±1.08	2.75±0.19	13.71±0.55	5.23±0.40	14.04±0.81
NIST2975 54 µg	6.07±0.72	15.38±1.34	3.80±1.37	14.07±1.70	5.27±0.86	13.97±1.36
NIST2975 162 µg	8.05±0.74	14.65±1.14	3.38±0.67	12.55±0.77	6.83±0.38	16.46±0.73
NIST1650[a] 18 µg	4.15±0.49	10.95±0.46	1.73±0.18	9.85±0.52	6.25±0.59	14.52±0.66
NIST1650[a] 54 µg	5.13±0.44	13.75±0.70	1.83±0.14	11.93±0.46	6.49±0.41	15.00±0.71
NIST1650[a] 162 µg	4.60±0.38	12.17±0.54	2.70±0.19	12.82±0.48	5.75±0.23	14.54±0.85
**Day 90**	**BAL cells**		**Lung tissue**		**Liver tissue**	
	% DNA in tail	***Tail length***	***% DNA in tail***	***Tail length***	***% DNA in tail***	***Tail length***
Vehicle control	4.32±0.55	13.33±0.72	3.65±0.59	13.99±0.83	5.30±0.37	14.92±0.24
Carbon black 54 µg	3.44±0.19	11.04±0.19	2.27±0.29	11.76±0.67	4.75±0.53	13.26±0.60
CAP 54 µg	3.67±0.21	11.39±0.32	3.93±0.71	14.01±1.04	5.15±0.70	12.98±0.82
JEP 54 µg	5.66±1.28	15.64±2.22(x)	3.02±0.52	11.11±0.70	4.73±0.33	14.31±0.75
NIST2975 162 µg	2.87±0.34	10.93±0.61	3.68±2.13	13.58±3.00	6.26±0.45	16.26±0.92

[a] NIST1650 data was included for comparison and obtained from a previously published study (Kyjovska et al. Mutagenesis 2015).
(*) = p<0.05 increase compared to **vehicle control, (x) – (xxxx)** = p<0.05 - p < 0.0001 increase compared to **CB 54 µg.** Mean±SEM (x 103).
